# Targeting G3BP1 Condensate Topology Promotes Stress Granule Assembly via m^6^A‐IGF2BP1 for Ischemic Stroke Rescue

**DOI:** 10.1002/advs.202514703

**Published:** 2025-11-20

**Authors:** Ling Li, Yong‐Dong Guo, Xiao‐Wen Zhang, Zhi‐Yong Du, Yu‐Qi Wang, Zhuo Yang, Qian‐Wei Luo, Fang‐Fang Zhuo, Tian‐Tian Wei, Zheng‐Ping Liu, Bo Han, Wei Yu, Pei‐Pei Zhang, Wei Zhou, Zhi‐Yuan Lu, Peng‐Fei Tu, Chun‐Hong Zheng, Ke‐Wu Zeng

**Affiliations:** ^1^ State Key Laboratory of Natural and Biomimetic Drugs School of Pharmaceutical Sciences Peking University Beijing 100191 China; ^2^ Department of Immunology School of Basic Medical Sciences Peking University Beijing 100191 China; ^3^ Key Laboratory of Remodeling‐Related Cardiovascular Diseases Ministry of Education National Clinical Research Center for Cardiovascular Diseases Beijing Institute of Heart Lung and Blood Vessel Disease Beijing Anzhen Hospital Capital Medical University Beijing 100029 China; ^4^ Shandong Engineering Research Center of New Sustained and Controlled Release Formulations and Drug Targeted Delivery Systems Shandong Academy of Pharmaceutical Sciences Jinan 250101 China; ^5^ School of Pharmacy/Key Laboratory of Xinjiang Phytomedicine Resource and Utilization Shihezi University Shihezi 832003 China; ^6^ Department of Biochemistry and Molecular Biology School of Basic Medical Sciences Peking University Health Science Center Beijing 100191 China; ^7^ State Key Laboratory of Natural Medicines School of Traditional Chinese Pharmacy China Pharmaceutical University Nanjing 211198 China; ^8^ School of Pharmaceutical Sciences & Institute of Materia Medica Shandong First Medical University & Shandong Academy of Medical Sciences Jinan 250117 China

**Keywords:** condensate topology, m^6^A, phase separation, ras‐GTPase‐activating protein SH3 domain‐binding protein 1 (G3BP1), stress granules (SGs)

## Abstract

Ras‐GTPase‐activating protein SH3 domain‐binding protein 1 (G3BP1) mediates stress granules (SGs) via phase separation. However, there is limited understanding of the allosteric mechanism and the identification of regulatory molecules. Here, we identify icariin (ICA), a small‐molecule inducer that promotes G3BP1‐driven biomolecular condensate formation, which effectively restructures SGs architecture. Moreover, we demonstrate that ICA interacts with the N‐terminal nuclear transport factor 2‐like (NTF2L) domain of G3BP1, inducing a conformational switch from “closed‐to‐open” that facilitates G3BP1 oligomerization and phase separation. Crucially, G3BP1 condensates recruit N^6^‐methyladenosine (m^6^A) reader insulin‐like growth factor 2 mRNA‐binding protein 1 (IGF2BP1) through topology‐selective scaffolding, establishing epitranscriptomic hubs that resolve proteotoxic stress via m^6^A‐dependent AMP‐activated protein kinase (AMPK)‐mitogen‐activated protein kinase (MAPK)‐glutathione peroxidase 4 (GPX4) signaling pathways. Strikingly, this chemical intervention shows translational potential, as ICA reduces cerebral infarct volume in ischemia models via G3BP1‐dependent SGs remodeling. Additionally, single‐nucleus transcriptomics identify *Fezf2*, *Pou3f1*, and *Kcnn2* neuronal subpopulations as mechanistically aligned responders. Furthermore, ischemic stroke patients reveal G3BP1–IGF2BP1–m^6^A axis within peripheral blood mononuclear cells. Taken together, this study redefines SGs as dynamically druggable epitranscriptomic processors for precision neuroprotection. In particular, a framework for leveraging biomolecular condensate topology in the development of next‐generation neurological therapeutics is offered.

## Graphical abstract

Our research reveals that small‐molecule icariin (ICA) directly binds Ras‐GTPase‐activating protein SH3 domain‐binding protein 1 (G3BP1), inducing conformational change and oligomerization. This interaction confers neuroprotection by recruiting insulin‐like growth factor 2 mRNA‐binding protein 1 (IGF2BP1) and modulating N^6^‐methyladenosine (m^6^A) modifications. Moreover, a distinct neuronal subset exhibits ICA sensitivity correlated with G3BP1 expression, suggesting therapeutic potential for stroke.

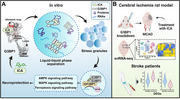

## 1 Introduction

Stress granules (SGs), membrane‐less organelles, play a critical role in the onset of human diseases.^[^ ^1^, ^2^, ^3^ ^]^ Ras‐GTPase‐activating protein SH3 domain‐binding protein 1 (G3BP1) is considered as a crucial regulator of SGs assembly, thereby mediating cellular stress response in various pathological processes.^[^ ^4^, ^5^, ^6^, ^7^ ^]^ In particular, abnormal G3BP1 phase separation is highly associated with multiple human diseases, including cancers, autoimmune disorders, neurodegeneration, and metabolic disorders.^[^ ^8^, ^9^, ^10^, ^11^, ^12^ ^]^ Consequently, targeting G3BP1 represents a promising strategy for cellular protection. In structure, G3BP1 consists of two domains and three intrinsically disordered regions (IDRs).^[^ ^13^, ^14^, ^15^ ^]^ The N‐terminal nuclear transport factor 2‐like (NTF2L) domain facilitates G3BP1 dimerization, stress granule nucleation, and recruitment of substrate proteins.^[^ ^16^, ^17^ ^]^ Moreover, the RNA recognition motif (RRM) recognizes and binds RNA to maintain mRNA homeostasis.^[^ ^13^, ^18^ ^]^ Meanwhile, the IDRs in G3BP1 are crucial in inducing weak multivalent protein–protein or protein–nucleic acid interactions to promote the liquid–liquid phase separation (LLPS) process.^[^ ^19^ ^]^ Therefore, the structural hierarchy, which integrates precise molecular recognition elements, positions G3BP1 as a distinct pharmacological target. Additionally, small‐molecules that specifically target these domains may provide innovative strategies for mitigating pathological phase transitions.

The presence of G3BP1 at the core of SGs during disease progression underscores its significance for developing new therapeutic strategies. Currently, the rational modulation of G3BP1 phase separation into SGs assembly is considered to yield valuable insights for disease treatment. Notably, there has been a surge of interest in the development of small‐molecule modulators for suppressing the phase separation of G3BP1.^[^ ^20^, ^21^, ^22^ ^]^ For instance, a previous study has demonstrated that small peptides (G3Ia and G3Ib) exhibit remarkable inhibitory effects on G3BP1 phase separation through specific binding to G3BP1, thereby offering potential therapeutic benefits for neurodegenerative disorders.^[^ ^19^ ^]^ Therefore, current therapeutic strategies primarily focus on inhibiting G3BP1 phase separation within pathological condensates. However, this approach paradoxically undermines an evolutionarily conserved cytoprotective program. Since the assembly of SGs represents an adaptive survival mechanism, it is imperative to explore whether the controlled enhancement of biomolecular condensation confers therapeutic benefits. In particular, the pharmacological activation of SGs formation remains a largely unexplored area in disease treatment.

In this study, we establish a high‐content phenotypic screening platform targeting G3BP1‐driven biomolecular condensates, leading to the discovery of icariin (ICA), a chemical probe that promote functional SGs assembly. Subsequently, we find that ICA bound to the NTF2L domain of G3BP1, inducing a topology‐restructuring conformational switch that facilitates G3BP1 oligomerization formation. Moreover, ICA demonstrates significant protective effects against various types of stress‐induced neuronal cell injury. Mechanically, G3BP1 induces the recruitment of insulin‐like growth factor 2 mRNA‐binding protein 1 (IGF2BP1) into SGs, orchestrating N^6^‐methyladenosine (m^6^A) modification of survival‐related mRNAs through coordinated regulation of the AMP‐activated protein kinase (AMPK)‐mitogen‐activated protein kinase (MAPK)‐glutathione peroxidase 4 (GPX4) signaling pathways. In vivo experiments using the middle cerebral artery occlusion (MCAO) model reveal that the G3BP1 knockdown significantly antagonizes the neuroprotective effects by ICA. In particular, single‐nucleus RNA sequencing identifies a unique subset of neurons positive for *Fezf2*, *Pou3f1*, and *Kcnn2* genes, characterized by pronounced correlation between ICA sensitivity and *G3BP1* gene expression. Furthermore, analysis of peripheral blood monocytes from ischemic stroke patients and healthy subjects reveals a significant association between G3BP1 expression and the incidence of cerebral ischemia.

Collectively, our study establishes the chemical engineering of G3BP1‐driven biomolecular condensates as a neuroprotective strategy. This pharmacologically tractable approach provides a roadmap for developing precision small‐molecule modulators that exploit phase‐separation dynamics to intercept ischemic neurodegeneration.

## 2 Results

### 2.1 Systematic Screening Identifies ICA as a G3BP1 Phase Separation Inducer

**Figure 1:**
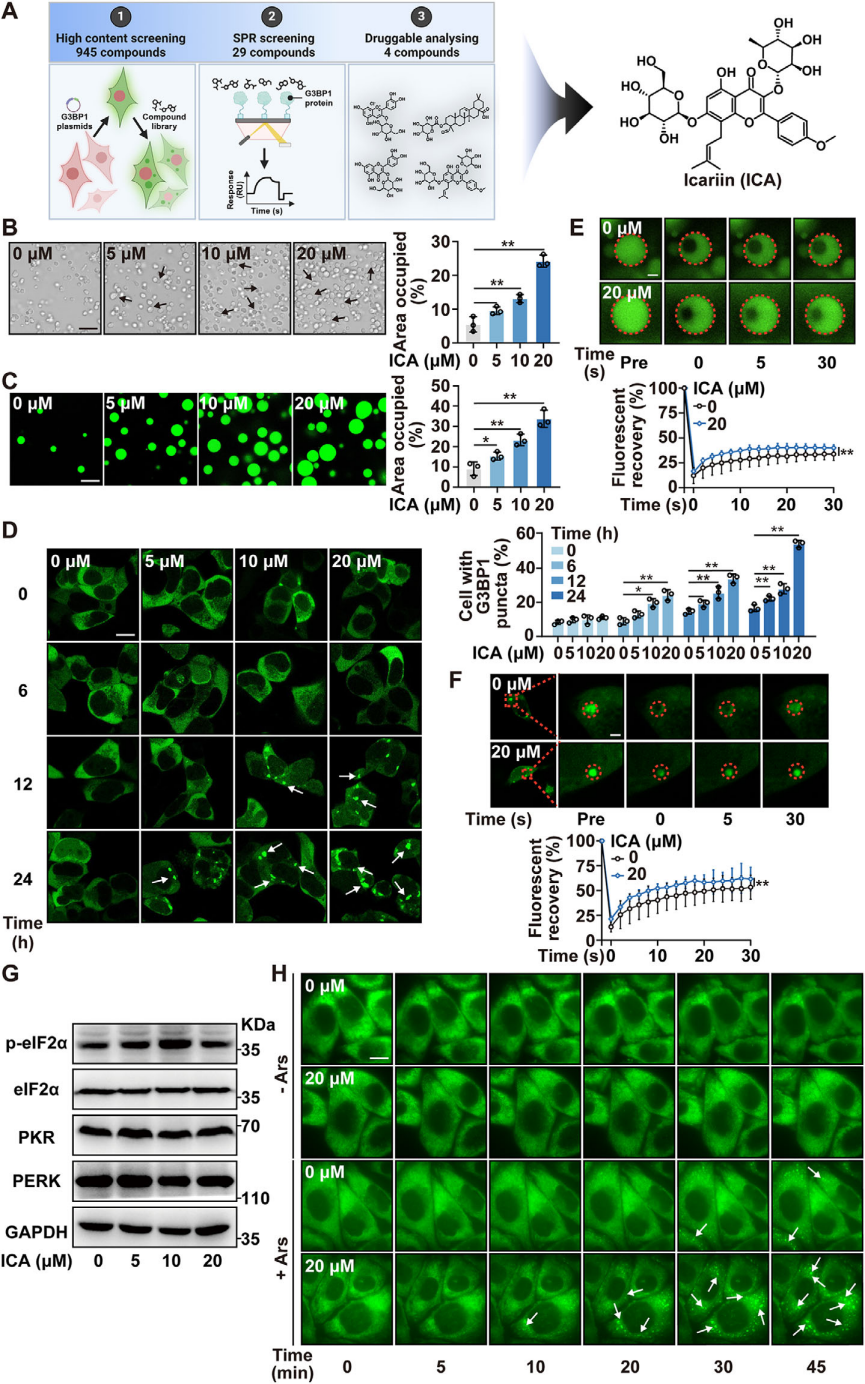
Systematic screening identifies ICA as a G3BP1 phase separation inducer. A) The screening process targeting G3BP1 and the chemical structure of ICA. B) Recombinant G3BP1 condensates formed with varying concentrations of ICA (0, 5, 10, and 20 µm) prior to RNA induction (100 ng µL^−1^) and 10% bovine serum albumin (BSA) as crowding agent (bar: 20 µm). C) Recombinant G3BP1–GFP condensates formed with varying concentrations of ICA (0, 5, 10, and 20 µm) prior to RNA induction (100 ng µL^−1^) and 10% BSA as crowding agent (bar: 20 µm). D) The HEK293T cells transfected with G3BP1–GFP plasmids were exposed to ICA in the time‐ and concentration‐dependent manners (bar: 20 µm). E) FRAP recovery analysis of G3BP1 condensates formed with ICA (20 µm) in vitro (bar: 1 µm). F) FRAP recovery analysis of G3BP1 condensates after transfecting with plasmids and treatment with ICA (20 µm) in cells (bar: 1 µm). G) Western blot analysis was performed to determine the protein expression of p‐eIF2α, eIF2α, PKR, and PERK in HEK293T cells. H) The U2OS cells overexpressed G3BP1–GFP were exposed to low‐dosage arsenite (100 µm) with or without ICA (20 µm) treatment (bar: 20 µm). Data are expressed as the mean ± standard deviation (SD) for 3 individual experiments. *p < 0.05, **p < 0.01.

Given the significant role of G3BP1 phase separation in the SGs formation,^[^ ^23^ ^]^ we aimed to identify small‐ molecules capable of inducing G3BP1 phase separation. Here, we established a screening system to specifically modulate G3BP1 phase separation (**Figure** **1**A). The *G3BP1* fusion gene plasmid, tagged with GFP, was transfected into HEK293T cells. Subsequently, the cells were treated with different compounds from our chemical library (945 compounds in total) at a concentration of 10 µm. The green fluorescent puncta were then quantified using high‐content screening technology. As a result, we identified 29 potential compounds that significantly increased the number of fluorescence spots in HEK293T cells (>2.5‐fold of control) without affecting fluorescence intensity (Figure S1A, Supporting Information). To further validate these findings, we performed a surface plasmon resonance (SPR) assay, which confirmed that four compounds exhibited strong binding to G3BP1, with a dissociation constant (*K*_D_) ranging between 3.1 and 8.9 µm, as shown in Figure S1B,C (Supporting Information). Among them, ICA attracted significant interest due to its unique chemical structure and extensive pharmacological activities reported in previous studies.^[^ ^24^ ^]^

Next, we decided to further elucidate the impact of ICA on G3BP1 phase separation. First, recombinant G3BP1 and G3BP1–GFP proteins were incubated with or without ICA in the presence of RNA at 25 °C to induce LLPS. As shown in Figure 1B,C, G3BP1 displayed the capacity to form liquid droplets, and ICA resulted in a concentration‐dependent enhancement of G3BP1 condensate formation. Subsequently, we transfected G3BP1–GFP plasmid into HEK293T cells. Our observations demonstrated that ICA led to a notable elevation of intracellular condensates, which correlated with both concentration and time (Figure 1D). Furthermore, we assessed the mobility of G3BP1 condensates by employing fluorescence recovery after photobleaching (FRAP). Our observation demonstrated a significant increase in the recovery percentage and a decrease in the half‐time of recovery (*T*_1/2_) upon ICA treatment. In vitro, the recovery percentage was ≈45% (*T*_1/2_ = 2.86 s), compared to 35% (*T*_1/2_ = 6.04 s) in the control (Figure 1E). Similarly, in cell experiments, the recovery percentage reached ≈65% (*T*_1/2_ = 5.40 s), whereas the control group exhibited 52% recovery (*T*_1/2_ = 7.55 s) (Figure 1F). In addition, the phosphorylation of eIF2α causes endoplasmic reticulum stress and is regarded as the upstream of SGs formation.^[^ ^25^ ^]^ Here, we observed that ICA did not affect the expression of p‐eIF2α, eIF2α, PKR, and PERK protein levels, indicating that the upstream mechanism of SGs formation was not modulated by ICA (Figure 1G). Since arsenite (Ars)‐induced SGs assembly in a short time is widely recognized,^[^ ^19^ ^]^ we found that ICA synergized with arsenite to promote G3BP1 aggregation, thereby facilitating SGs formation under low‐dose, short‐term arsenite treatment (Figure 1H). Thus, ICA served as an essential initiator for nucleation of G3BP1‐positive SGs. In summary, our study identified ICA as a small‐molecule that effectively facilitated SGs formation through G3BP1 phase separation.

### 2.2 ICA Directly Binds to the NTF2L Domain of G3BP1

**Figure 2:**
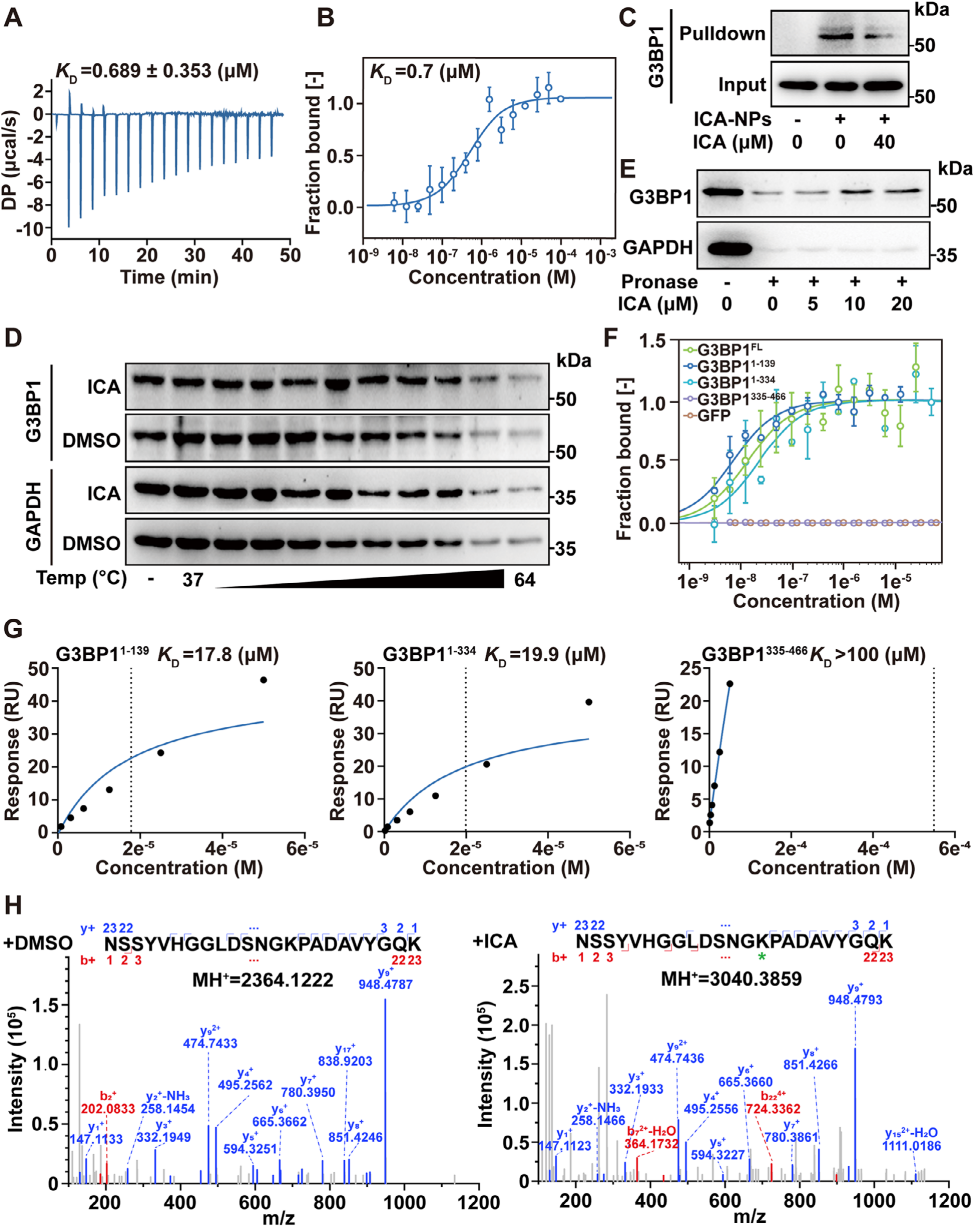
ICA directly binds to the NTF2L domain of G3BP1. A,B) ITC and MST analysis of ICA binding to recombinant G3BP1 protein. C) Pull‐down analysis of ICA binding to G3BP1 in cell lysate. D) ICA promoted the temperature‐dependent stabilization of G3BP1 through CETSA. E) ICA enhanced G3BP1 resistance against pronase through DARTS. F) MST analysis of ICA binding to full‐length or different variants of G3BP1 protein. G) SPR analysis of ICA binding to different variants of G3BP1 protein. H) LC–MS/MS analysis of covalently modified peptide between ICA with G3BP1^1–139^. Data are expressed as the mean ± SD for 3 individual experiments. *p < 0.05, **p < 0.01.

Recent studies have elucidated the capacity of small‐molecules to regulate LLPS by directly binding to specific protein domains.^[^ ^20^, ^26^, ^27^ ^]^ First, we attempted to validate the direct interaction between ICA and G3BP1. Isothermal titration calorimetry (ITC) analysis revealed that ICA bound to G3BP1 with a *K*_D_ of 0.689 ± 0.353 µm. The negative free energy change (Δ*G* = −8.41 kcal mol^−1^) confirmed the spontaneity and favorability of the interaction, with thermodynamic constant Δ*H* and −*T*Δ*S* values of −118 ± 13.1 and 110 kcal mol^−1^, respectively (**Figure** **2**A; Figure S2A, Supporting Information). This result was supported by microscale thermophoresis (MST) analysis (*K*_D_ = 0.7 µm) (Figure 2B). Subsequently, we synthesized ICA‐cross‐linked nanoparticles (ICA‐NPs) as previously described (Figure S2B, Supporting Information).^[^ ^28^ ^]^ Pull‐down assays demonstrated that ICA‐NPs effectively captured G3BP1 from cell lysates, and this binding was competitively inhibited by free ICA (Figure 2C; Figure S2C, Supporting Information). Furthermore, cellular thermal shift assay (CETSA) suggested that ICA enhanced the thermal stability of G3BP1 (Figure 2D; Figure S2D, Supporting Information). Meanwhile, drug affinity responsive target stability (DARTS) experiment demonstrated that ICA stabilized G3BP1 by increasing its resistance to proteolysis (Figure 2E; Figure S2E, Supporting Information). Although G3BP2 shares high sequence homology with G3BP1 and is also a core component of SGs formation,^[^ ^14^ ^]^ both CETSA and DARTS assays indicated that ICA showed no significant effect on G3BP2 protein (Figure S2F, G, Supporting Information). Taken together, these results indicated a specific interaction between ICA and G3BP1, rather than G3BP2.

The G3BP1 protein comprises two principal domains, the NTF2L and RRM domains.^[^ ^4^, ^16^ ^]^ To identify the ICA‐binding domain, we designed and generated three truncations of G3BP1: G3BP1^1–139^ (NTF2L domain), G3BP1^1–334^ (NTF2L and IDR1/2 domains), and G3BP1^335–466^ (RRM and IDR3 domains) (Figure S2H, Supporting Information). To further evaluate the selectivity of ICA for different G3BP1 truncations, we performed MST experiments using cell lysates overexpressing G3BP1 or its truncated variants. The results showed that the G3BP1^1–139^ and G3BP1^1–334^ exhibited higher affinity with ICA compared to the G3BP1^335–466^ (Figure 2F). Similarly, we purified recombinant truncations and SPR experiments revealed that the binding of ICA to G3BP1^1–139^ (*K*_D_ = 17.8 µm) and G3BP1^1–334^ (*K*_D_ = 19.9 µm) was significantly stronger than to G3BP1^335–466^ (*K*_D_ > 100 µm) (Figure 2G). Given that the G3BP1 becomes more stabilized during proteolysis following ICA treatment, we attempted to use liquid chromatography–tandem mass spectrometry (LC–MS/MS) analysis to identify the peptides protected by ICA. Notably, one peptide (residues 37–59), located in the NTF2L domain, remained stable following enzymatic digestion (Figure S2I, Supporting Information). To investigate the specific binding domain of G3BP1 by ICA, we conducted mass spectrometry analysis and identified a covalent modification site at lysine 50 (K50) in the NTF2L domain of G3BP1 (Figure 2H). Here, we proposed that ICA initially engages in noncovalent binding within a pocket proximal to Lys50, driven by complementary interactions. This docking geometry positions the electrophilic α, β‐unsaturated ketone moiety of ICA toward the nucleophilic ε‐amino group (─NH_2_) of Lys50, thereby enabling covalent adduct formation (Figure S2J, Supporting Information). Therefore, these results provided evidence that ICA preferred to bind to NTF2L domain of G3BP1.

### 2.3 ICA Induces G3BP1 Phase Separation via the NTF2L Domain Oligomerization

**Figure 3:**
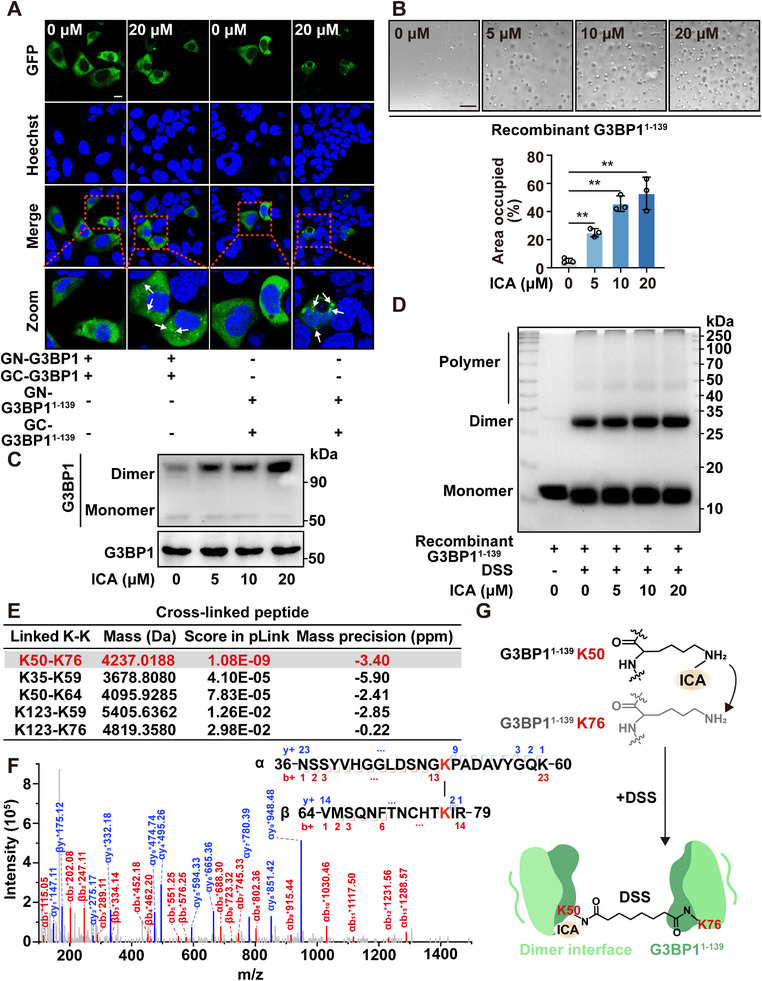
ICA induces G3BP1 phase separation via the NTF2L domain dimerization. A) ICA promoted intracellular G3BP1 and G3BP1^1–139^ dimerization or oligomerization by bimolecular fluorescence complementation (BiFC) assay (bar:20 µm). B) Recombinant G3BP1^1–139^ condensates formed with various concentrations of ICA (5, 10, and 20 µm) (bar: 20 µm). C) The HEK293T cells were exposed to ICA (5, 10, and 20 µm), and the dimerization of G3BP1 was assessed by native polyacrylamide gel electrophoresis. D) The recombinant G3BP1^1–139^ proteins were incubated with ICA (5, 10, and 20 µm) followed by cross‐link with DSS. The protein was separation by sodium dodecyl sulfate– polyacrylamide gel electrophoresis (SDS‐PAGE) electrophoresis and visualization by coomassie brilliant blue staining. E) Cross‐link analysis of recombinant G3BP1^1–139^ incubated with ICA (20 µm). Protein cross‐links were analyzed with pLink. F) High‐resolution MS/MS spectrum of the inter G3BP1^1–139^ cross‐link between K50 and K76. G) The cross‐link mechanism of K50–K76 in G3BP1 dimer modeling. Data are expressed as the mean ± SD for 3 individual experiments. *p < 0.05, **p < 0.01.

Previous research has indicated that the NTF2L domain of G3BP1 can form homodimers or interact with other proteins to facilitate phase separation.^[^ ^7^ ^]^ Consequently, we evaluated the impact of ICA on G3BP1 dimerization or oligomerization using a bimolecular fluorescence complementation (BiFC) reporter system in cells.^[^ ^29^ ^]^ Here, we fused G3BP1 and G3BP1^1–139^ to the split GFP fluorophores GN (residues 1–155) or GC (residues 156–238) at the N‐terminal.^[^ ^30^ ^]^ ICA treatment resulted in a notable enhancement of the green fluorescent puncta observed, alongside transfections (**Figure** **3**A). Thus, we proposed that the homodimer of NTF2L domain was induced by ICA, as a core for the formation of droplets. Additionally, our findings indicated that NTF2L domain alone was sufficient to undergo phase separation in the presence of ICA, while other G3BP1 variants did not exhibit phase separation under identical conditions (Figure 3B; Figure S3A,B, Supporting Information). Thus, this observation underscored the pivotal role of the NTF2L domain in ICA‐induced phase separation and SGs formation.

To investigate the oligomeric state of G3BP1, we performed native polyacrylamide gel electrophoresis. Our finding suggested that ICA promoted the dimerization of G3BP1 while showing no substantial effect on protein expression (Figure 3C). Furthermore, chemical cross‐linking with disuccinimidyl suberate (DSS) was employed to examine G3BP1 oligomerization.^[^ ^31^ ^]^ This analysis revealed that ICA significantly enhanced the oligomerization of G3BP1^1–139^, as evidenced by an increased presence of bands larger than 28 kDa (Figure 3D). Additionally, we utilized chemical cross‐linking mass spectrometry coupled with pLink software to elucidate intermolecular interactions between G3BP1^1–139^ following ICA treatment (Figure 3E). Our data revealed that the MS signal intensities of Lys50 (located at residues 37–59) and Lys76 (located at residues 65–78) were significantly greater than those observed in the dimethyl sulfoxide (DMSO) treatment (Figure 3F,G). Meanwhile, our findings suggested that the two amino acid residues represented spatial proximity sites during ICA‐induced G3BP1 oligomer formation. Additionally, we also identified four additional lysine–lysine cross‐links, including Lys35–Lys59, Lys50–Lys64, Lys123–Lys59, and Lys123–Lys76, located at the reported dimer interface or in close spatial proximity. However, we found that these peptides did not show significant differences in mass spectrometry intensity after ICA treatment and were considered as nonspecific cross‐links. We then performed dynamic light scattering (DLS) analysis to precisely characterize the size distribution of G3BP1 aggregates in the presence and absence of ICA. Our results indicated a concentration‐dependent increase in hydrodynamic radius upon ICA treatment (Figure S3C, Supporting Information). Taken together, our results demonstrated that ICA promoted the formation of G3BP1 dimers or oligomer via the NTF2L domain.

### 2.4 Allosteric Regulation Drives the LLPS of G3BP1 through a “Close‐to‐Open” Pattern

**Figure 4:**
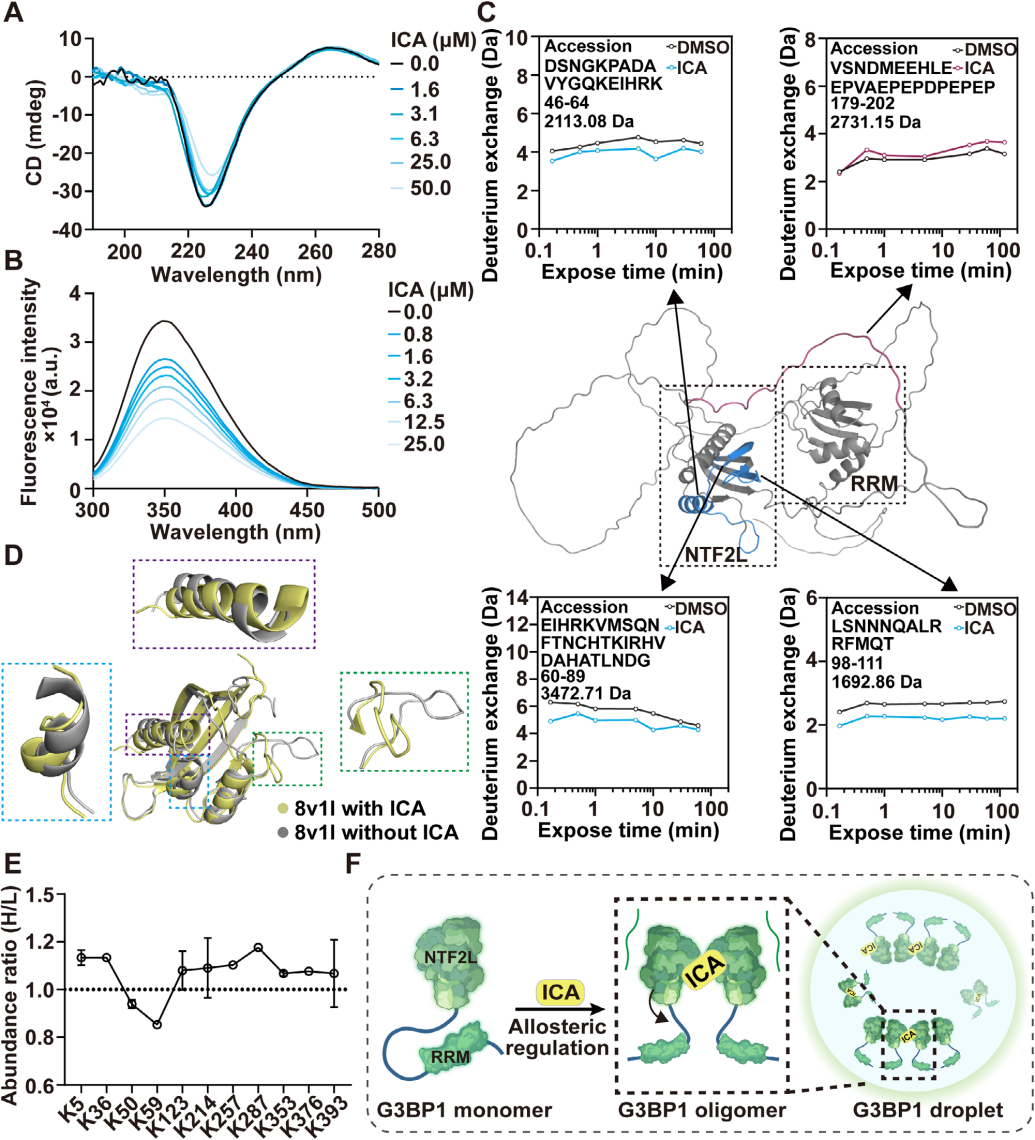
Allosteric regulation drives the LLPS of G3BP1 through a “close‐to‐open” pattern. A) CD spectra analysis for ICA‐induced G3BP1 conformational change. B) Tryptophan fluorescence quenching assay for detecting ICA‐induced G3BP1 conformational change. C) Hydrogen–deuterium exchange (HDX) profiles of G3BP1 conformational change after treatment with ICA. Peptides with different H/D exchange in G3BP1 are highlighted in pink (H/D exchange increase) or blue (H/D exchange decrease). D) 3D model of ICA‐induced G3BP1 allosteric regulation in molecular dynamics simulation (PDB: 8v1l). E) Quantitative lysine reactivity profiling strategy to evaluate the reactivity of lysine residues. F) The proposed allosteric regulation mechanism of G3BP1 by ICA. Data are expressed as the mean ± SD for 3 individual experiments.

Previous studies have demonstrated that conformational changes in G3BP1 can influence the binding affinity to RNA and proteins, we then hypothesized that ICA may affect the protein conformation of G3BP1 through binding NTF2L domain. Circular dichroism (CD) spectroscopy revealed a decrease in α‐helix and β‐sheet content upon ICA treatment (**Figure** **4**A; Figure S4A, Supporting Information). Meanwhile, tryptophan fluorescence scanning showed a concentration‐dependent decrease in G3BP1 fluorescence upon treatment with ICA (Figure 4B). These results demonstrated that ICA binding triggered conformational changes in the G3BP1 protein. We subsequently employed hydrogen–deuterium exchange mass spectrometry (HDX‐MS) to investigate the allosteric mechanism of full‐length G3BP1.^[^ ^32^ ^]^ As shown in Figure 4C and Figure S4B (Supporting Information), treatment with ICA significantly decreased hydrogen/deuterium exchange levels in three specific peptides within the NTF2L domain, peptides 46–64 (DSNGKPADAVYGQKEIHRK) located between the αIII‐helix and βI fold, peptides 60–89 (EIHRKVMSONFTNCHTKIRHVDAHATLNDG) and 98–111 (LSNNNQALRRFMQT) positioned at the dimer interface. As ICA binds to Lys50, which was embedded within the hydrophobic cavity formed by αIII‐helix and βI fold, facilitated the spatial proximity of the Lys76 residue in the adjacent monomer, thereby reducing solvent accessibility of these peptide. The decreased exchange rate of peptides 98–111 was likely due to the enhanced interactions at the dimer interface (such as polar interactions) following ICA treatment, which stabilized the dimer structure and reduced solvent exposure. Furthermore, a modest increase in hydrogen/deuterium exchange levels was observed for 179–202 (VSNDMEEHLEEPVAEPEPDPEPEP) peptides located in the IDR1 region. In addition, the IDR1 region (containing peptides 179–202) has been reported to inhibit oligomerization of G3BP1 dimers by binding to NTF2L.^[^ ^7^ ^]^ Here, we observed an increase in the exchange levels within the IDR1 region, indicating potential dissociation of the IDR1 domain from the NTF2L domain. Meanwhile, this dissociation likely enhanced solvent exposure of IDR1, alleviating the inhibitory effect on G3BP1 oligomerization, thereby facilitating oligomer formation. To further elucidate the allosteric regulatory mechanism of ICA on G3BP1, molecular dynamics (MD) simulations were performed (Figure 4D; Figure S4C,D, Supporting Information). As expected, the simulations revealed significant inward positioning of residues 44–51 relative to the αIII‐helix of NTF2L, which was consistent with the previous HDX results. Besides, our results revealed additional conformational shifts in α‐helices I and II. Furthermore, the thermodynamic analysis revealed a Gibbs free energy (Δ*G*) of −108.84 kcal mol^−1^ (with ICA) comparing to −93.62 kcal mol^−1^ (without ICA), indicating increased stability and compactness of the complex.

In addition, we also employed an active isotope dimethyl labeling assay to quantify lysine reactivity and validate the mechanism of G3BP1 conformational changes. Quantitative proteomics reveal heavy/light dimethyl isotope ratios for lysine residues.^[^ ^33^, ^34^ ^]^ A total of 11 lysines exhibited dimethyl modification. Notably, ICA treatment increased dimethylation of Lys287 (located in IDR2) while reducing modification at Lys50 and Lys59 in the NTF2L domain (Figure 4E; Figure S4E, Supporting Information). Notably, the decreased dimethylation of Lys50 and Lys59 might be due to the inward collapse of the NTF2L region following ICA binding to K50, consistent with the HDX and MD results. The integrated analysis of combined ^1^H–^15^N chemical shift perturbations enabled precise tracking of chemical shift trajectories, compelling evidence for site‐specific alterations in amino acid microenvironments. Our data demonstrated that ICA‐induced chemical shift perturbations in three residues of G3BP1^1–139^ (labeled in Boxes 1–3), accompanied by the disappearance of original cross‐peaks and the emergence of new signals (labeled in Boxes 4 and 5), indicating a potential conformational change (Figure S4F, Supporting Information). Therefore, these findings revealed that G3BP1 underwent conformational changes upon ICA treatment, resulting in a spatial open pattern (Figure 4F).

### 2.5 ICA Demonstrates a Cell‐Protective Capability Against Various Stress Injuries

**Figure 5:**
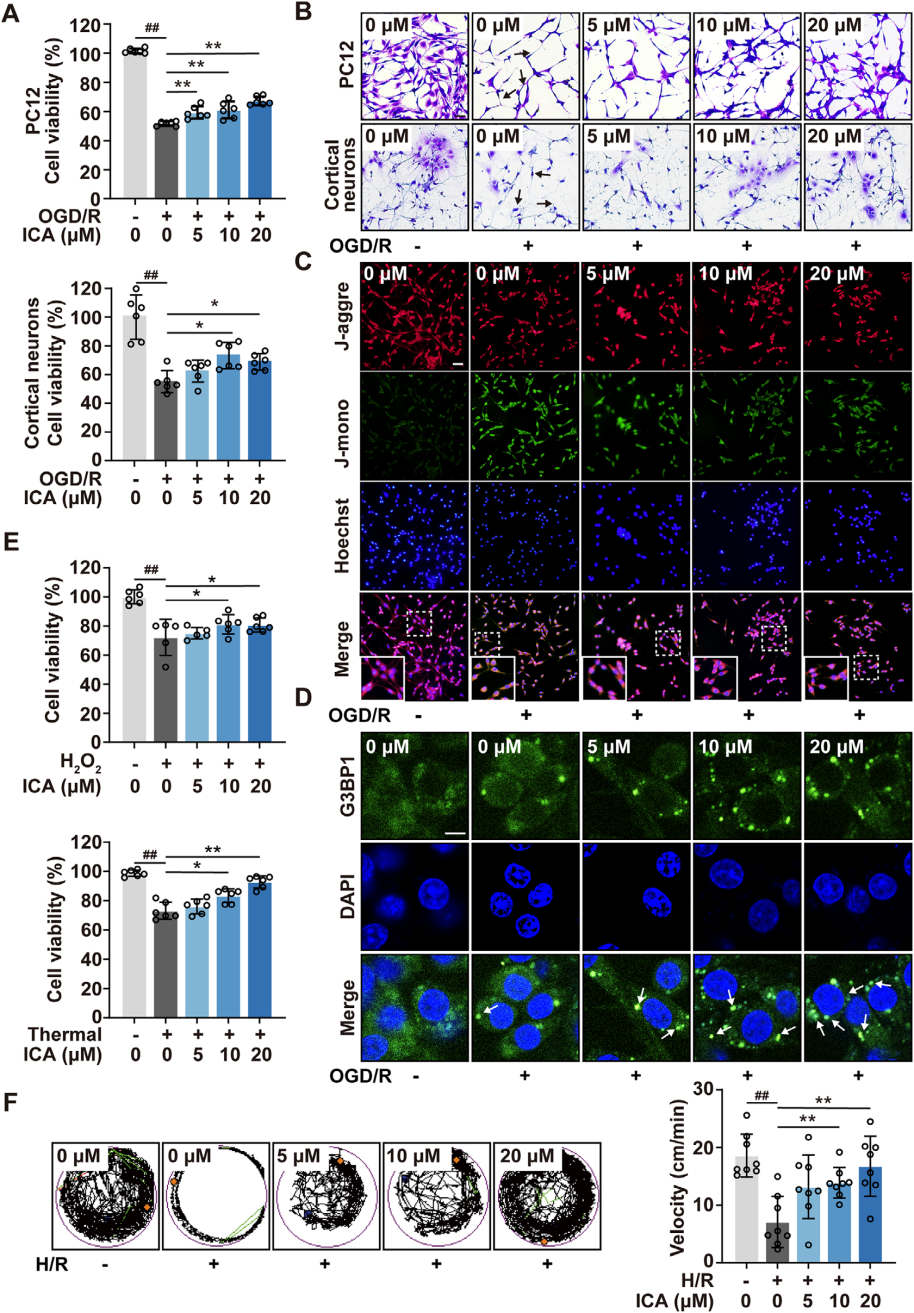
ICA demonstrates a cell‐protective capability against various stress injuries. A) The MTT assay of PC12 cells and cortical neurons following OGD/R injury upon ICA treatment. B) ICA protected cells against OGD/R injury by crystal violet staining (bar: 50 µm). C) ICA decreased OGD/R‐induced PC12 cells apoptosis by JC‐1 staining. (bar: 50 µm). D) SGs formation in PC12 cells underwent OGD/R stimulation with or without the presence of ICA using immunofluorescence (bar: 20 µm). E) The MTT assay was assessed for cell viability in PC12 cells induced with H_2_O_2_/thermal with or without ICA treatment. F) Neuroprotective effect of ICA in a zebrafish hypoxia/reoxygenation (H/R) injury model. Data are expressed as the mean ± SD for 3–8 individual experiments. *p < 0.05, **p < 0.01; ^#^ *p* < 0.05, ^##^ *p* < 0.01.

Previous studies have indicated that stress injuries can induce cells to spontaneously form protective SGs, prompting us to speculate that rational promoting the SGs formation may be a valuable cellular protection strategy.^[^ ^35^, ^36^ ^]^ To this end, we examined the protective efficacy of ICA against neuronal injury induced by ischemia. The efficacy of ICA in preventing cell death was demonstrated in PC12 cells and primary cultured cortical neurons subjected to oxygen–glucose deprivation followed by reoxygenation (OGD/R) (**Figure** **5**A). Furthermore, ICA treatment alone exhibited no cytotoxic effects (Figure S5A, Supporting Information). Consistent with these results, crystal violet staining showed that ICA rescued cell morphology, thereby maintaining viability (Figure 5B; Figure S5B, Supporting Information). Then, JC‐1 and AO/EB staining revealed that the percentage of apoptotic cells increased during OGD/R insult, which was reversed following ICA treatment (Figure 5C; Figure S5C, Supporting Information). We utilized DCFH‐DA and MitoSOX Red staining to detect the levels of intracellular hydrogen peroxide and mitochondrial superoxide anions. The results indicated that the increases in hydrogen peroxide and mitochondrial superoxide anions induced by OGD/R modeling were effectively reversed by ICA (Figure S5D, Supporting Information). Furthermore, G3BP1 immunofluorescence (IF) experiments showed that ICA significantly promoted SGs formation in cells under OGD/R (Figure 5D). These results indicated that ICA has the ability to induce the formation of SGs in stress conditions, suggesting a potential mechanism by which ICA may confer cellular protection.

To validate our findings under diverse stress conditions, we employed the chemical and physical stimulations (hydrogen peroxide and heat shock treatments) to construct cellular stress models. Then, subsequent investigation revealed that ICA significantly increased cell viability in these injury models, demonstrating a broad cytoprotective effect (Figure 5E). Furthermore, we investigated the neuroprotective effects of ICA using a zebrafish model of hypoxia–reoxygenation injury. ICA treatment significantly amelioration hypoxia‐induced behavioral deficits, demonstrating an obvious in vivo neuroprotection (Figure 5F). In summary, our data indicated that ICA exhibited a wide range of cellular protective effects potentially through inducing the formation of SGs.

### 2.6 G3BP1 Condensates Recruit IGF2BP1 to Modulate RNA Stability in m^6^A‐Dependent Manner

**Figure 6:**
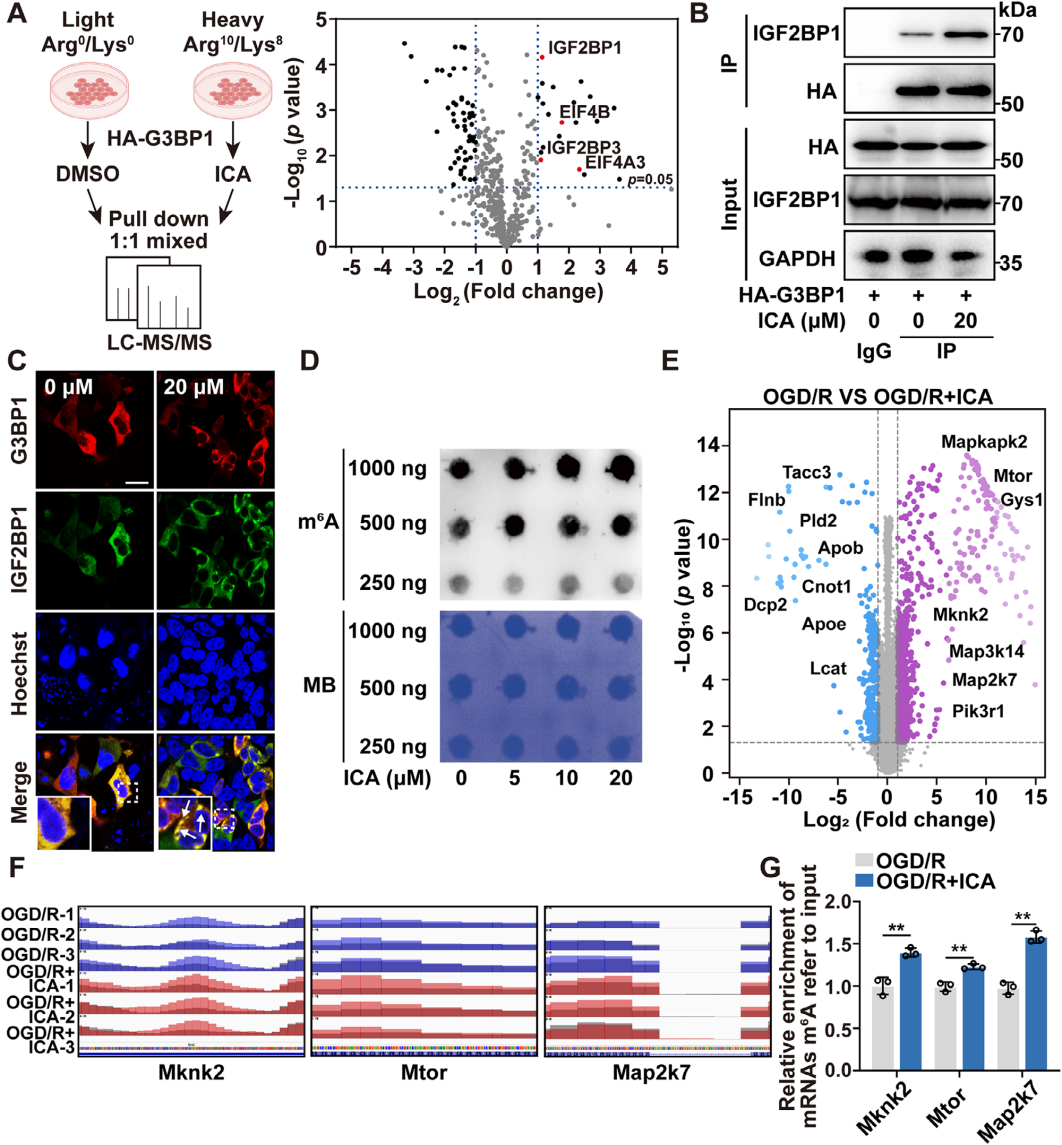
G3BP1 condensates recruit IGF2BP1 to modulate RNA stability in m^6^A‐dependent manner. A) Workflow of co‐immunoprecipitation (co‐IP) experiment combined with LC–MS/MS for proteomic analysis of G3BP1 interacting proteins following ICA treatment. B) Co‐IP assay with HA‐tagged beads, followed by anti‐IGF2BP1 antibody. C) Immunofluorescence analysis for the colocalization of G3BP1 (red) and IGF2BP1 (green) (bar: 20 µm). D) The dot blot assay for the ICA treatment on the global m^6^A abundance. E) The volcano plot of significant differentially expressed m^6^A peaks in OGD/R (*n* = 3) and OGD/R + ICA (*n* = 3) group. F) Visualization of the differential m^6^A abundance peak in transcript in OGD/R and OGD/R + ICA group. G) MeRIP‐quantitative‐polymerase chain reaction (qPCR) analysis of specific m^6^A‐modified RNAs in OGD/R and OGD/R + ICA group. Data are expressed as the mean ± SD for 3 individual experiments. *p < 0.05, **p < 0.01.

G3BP1 plays a pivotal role in SGs formation by interacting with various essential functional proteins to regulate RNA stability.^[^ ^37^ ^]^ To identify potential G3BP1‐interacting proteins, we performed stable isotope labeling with amino acids in cell culture (SILAC)‐based proteomic experiments using HA–G3BP1 as bait. Subsequently, we performed mass spectrometry analysis to detect the interacting proteins from the G3BP1 pulldown experiment (**Figure** **6**A). We subsequently constructed the protein–protein interaction (PPI) network, highlighting the increased interactions with G3BP1 following treatment with ICA (Figure S6A, Supporting Information). The identified proteins included eukaryotic translation initiation factors (eukaryotic translation initiation factor 4B (EIF4B) and eukaryotic translation initiation factor 4A3 (EIF4A3)) and RNA‐binding proteins (insulin‐like growth factor 2 mRNA‐binding protein 1 (IGF2BP1) and insulin‐like growth factor 2 mRNA‐binding protein 3 (IGF2BP3)), which have been previously reported as major components involved in the assembly of SGs. Of note, IGF2BPs, functioning as N⁶‐Methyladenosine (m^6^A) readers, play crucial roles in stabilizing and storing target mRNAs.^[^ ^38^ ^]^ In particular, our further analysis revealed that ICA exhibited a pronounced effect in enhancing the interaction between IGF2BP1 and G3BP1 in cells, as well as their colocalization within stress granules (Figure 6B,C). We speculated that IGF2BPs may be a key participant in the assembly of SGs formed by G3BP1.

Considering that IGF2BPs are widely recognized as m^6^A readers that regulate the stability of RNA, we then explored the potential downstream mechanisms mediated by ICA.^[^ ^38^ ^]^ Through the m^6^A dot blot assays, it was observed that the global abundance of m^6^A increased in a dose‐ and time‐dependent manner following treatment with ICA (Figure 6D; Figure S6B, Supporting Information). Previous studies have shown that the m^6^A modification is highly abundant in the brain, and its dysregulation contributes to in brain development abnormalities and neurodegenerative diseases. Notably, global m^6^A exhibits a biphasic pattern following OGD/R injury that transient elevation during acute oxygen–glucose deprivation was subsequently attenuated with extended reperfusion.^[^ ^39^ ^]^ Then, we employed methylated RNA immunoprecipitation sequencing (MeRIP‐Seq) to characterize m^6^A methylation profiles in OGD/R and OGD/R + ICA group. We identified an average of 17 768 m^6^A peaks in OGD/R injury group and 18 982 m^6^A peaks in OGD/R + ICA group. Of these, 16 086 were shared, while 1682 were unique to OGD/R group and 2896 unique to OGD/R + ICA group (Figure S6C, Supporting Information). Of note, the increased peak number in the OGD/R + ICA group indicated enhanced m^6^A modification upon ICA treatment. In addition, our results indicated that the m^6^A peaks were predominantly enriched in the coding sequences and 3′ untranslated regions (Figure S6D,E, Supporting Information). Furthermore, ICA treatment resulted in 446 upregulated m^6^A peaks and 184 downregulated m^6^A peaks, from 312 and 154 mRNAs (Figure 6E).

We next examined the potential roles of mRNAs with altered m^6^A methylation via Kyoto Encyclopedia of Genes and Genomes (KEGG) analyses. The results indicated that the mRNAs with upregulated m^6^A peaks were enriched in Tight junction, AMPK, and ErbB signaling pathways (Figure S6F, Supporting Information). Meanwhile, the increased m^6^A modifications of the genes such as *Mknk2*, *Mtor*, and *Map2k7* were highly associated with SGs formation (Figure 6F,G). In summary, these findings provided evidence that ICA facilitated the recruitment of IGF2BP1 to G3BP1, which played a critical role in the SGs formation for regulating m^6^A modification.

### 2.7 G3BP1 Regulates Neuronal Survival via AMPK–MAPK–GPX4 Signaling Axis

**Figure 7:**
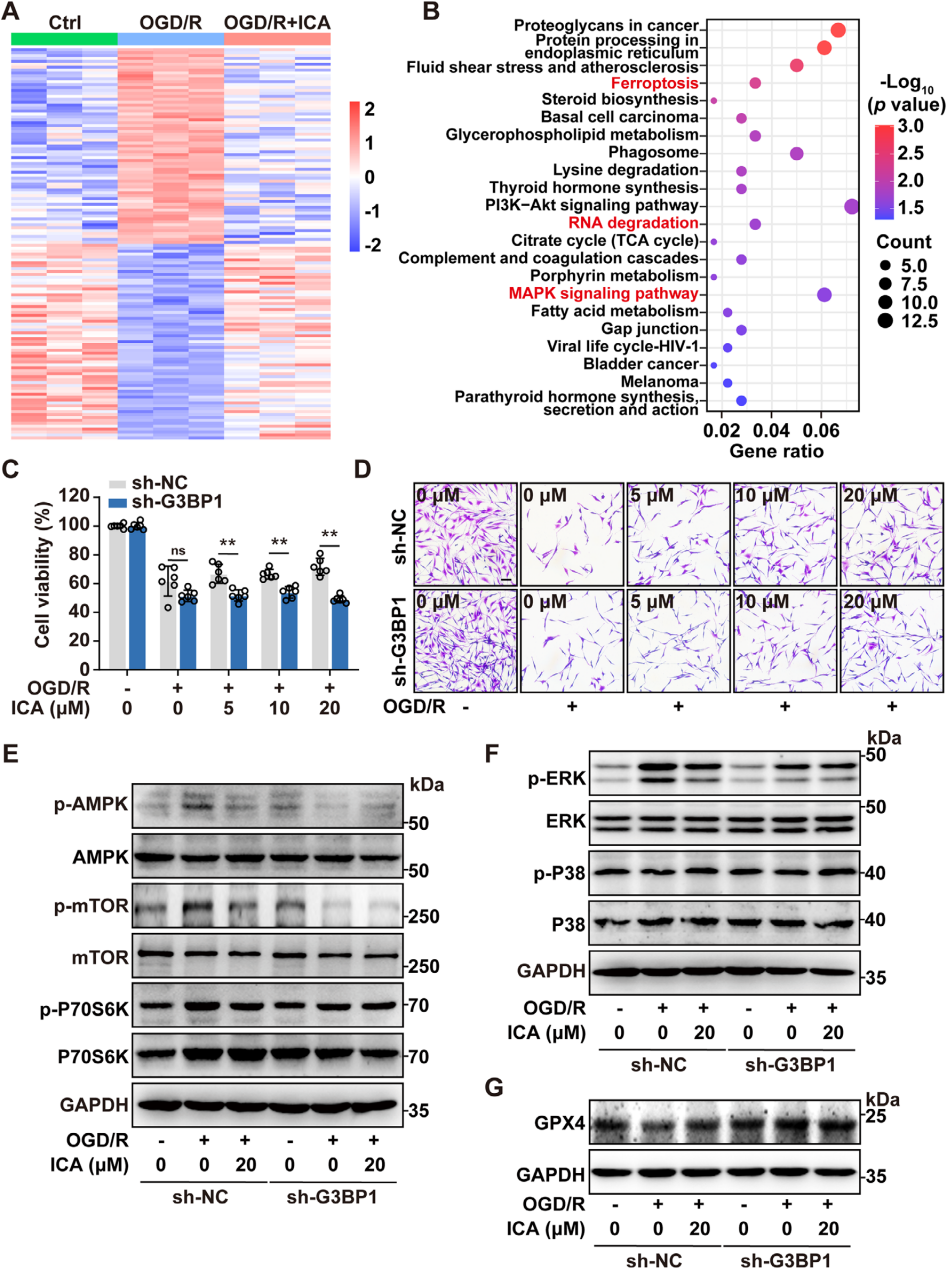
G3BP1 regulates neuronal survival via AMPK–MAPK–GPX4 signaling axis. A) Heatmap represented the levels of differentially expressed genes from the control (*n* = 3), OGD/R (*n* = 3), and OGD/R + ICA (*n* = 3) groups after ICA treatment. B) KEGG analysis revealed the potential role of DEGs from the control, OGD/R, and OGD/R + ICA groups after ICA treatment. C) MTT assay detected the cell viability after G3BP1 knockdown with or without ICA treatment follow by OGD/R. D) G3BP1 knockdown reversed the effect of ICA against OGD/R injury by crystal violet staining (bar: 50 µm). E–G) Western blot analysis of p‐AMPK, AMPK, p‐mTOR, mTOR, p‐P70S6K, P70S6K, p‐P38, P38, p‐ERK, ERK, and GPX4 protein levels after G3BP1 knockdown with or without ICA treatment during OGD/R. GAPDH served as a loading control. Data are expressed as the mean ± SD for 3–6 individual experiments. *p < 0.05, **p < 0.01; ns no significance.

To investigate the association between m^6^A methylation and gene expression, we then carried out RNA‐seq analysis on control group, OGD/R group, and OGD/R + ICA group. A total of 3164 differentially expressed genes (DEGs) were identified between OGD/R and control group. 701 DEGs were detected between OGD/R + ICA and OGD/R group (not shown in paper). The heatmaps showed the significant modulation of DEGs after ICA treatment. Moreover, GSEA and KEGG pathway analysis revealed that these DEGs were closely related to RNA degradation, MAPK signaling, and ferroptosis pathways (**Figure** **7**A,B; Figure S7A, Supporting Information). Notably, these pathways exhibited a close association with the SGs formation such as *Steap3*, *Fgfr1*, *Mknk2*, *Hspa9*, and *Hmox1*. To investigate the role of G3BP1 in the protective effects of ICA against OGD/R‐induced injury, we utilized a G3BP1‐specific lentivirus short hairpin RNA (sh‐G3BP1) lentivirus for G3BP1 knockdown (Figure S7B, Supporting Information). The results demonstrated that the protective effects of ICA against OGD/R were significantly attenuated in the knockdown group by MTT and crystal violet assays (Figure 7C,D). To evaluate the involvement of PDE5 in the neuroprotective effects of ICA, we examined PC12 cell viability following ICA treatment combined with either PDE5 inhibitor Sildenafil (Sil) or PDE5 siRNA knockdown. As shown in Figure S7C, D (Supporting Information), neither pharmacological inhibition by Sil nor genetic knockdown of PDE5 significantly modified the protective effect of ICA against OGD/R‐induced injury. Since AMPK pathway plays a pivotal role in regulating RNA degradation during cerebral ischemia,^[^ ^40^, ^41^, ^42^ ^]^ we next examined the proteins involved in the AMPK signaling cascade by western blot. As shown in Figure 7E, OGD/R activated the AMPK pathway, an effect that was reversed by ICA treatment. However, this protection was abolished following the knockdown of G3BP1. Similarly, MAPK signaling, enriched in KEGG analysis, showed reduced p‐ERK expression upon ICA treatment regardless of OGD/R exposure, which was reversed by sh‐G3BP1 (Figure 7F). Additionally, ICA upregulated GPX4, a key ferroptosis regulator responsible for reducing lipid hydroperoxides,^[^ ^43^ ^]^ after OGD/R, and this upregulation was suppressed by G3BP1 knockdown (Figure 7G). Consistent results were also observed with IGF2BP1‐specific short interfering RNA (si‐IGF2BP1) transfection (Figure S7E–H, Supporting Information). Collectively, these findings suggested that ICA mitigated OGD/R‐induced cell injury by targeting G3BP1 to facilitate SGs formation, thereby modulating the AMPK–MAPK–GPX4 signaling axis.

### 2.8 G3BP1 as a Therapeutic Target in the MCAO Rat Model

**Figure 8:**
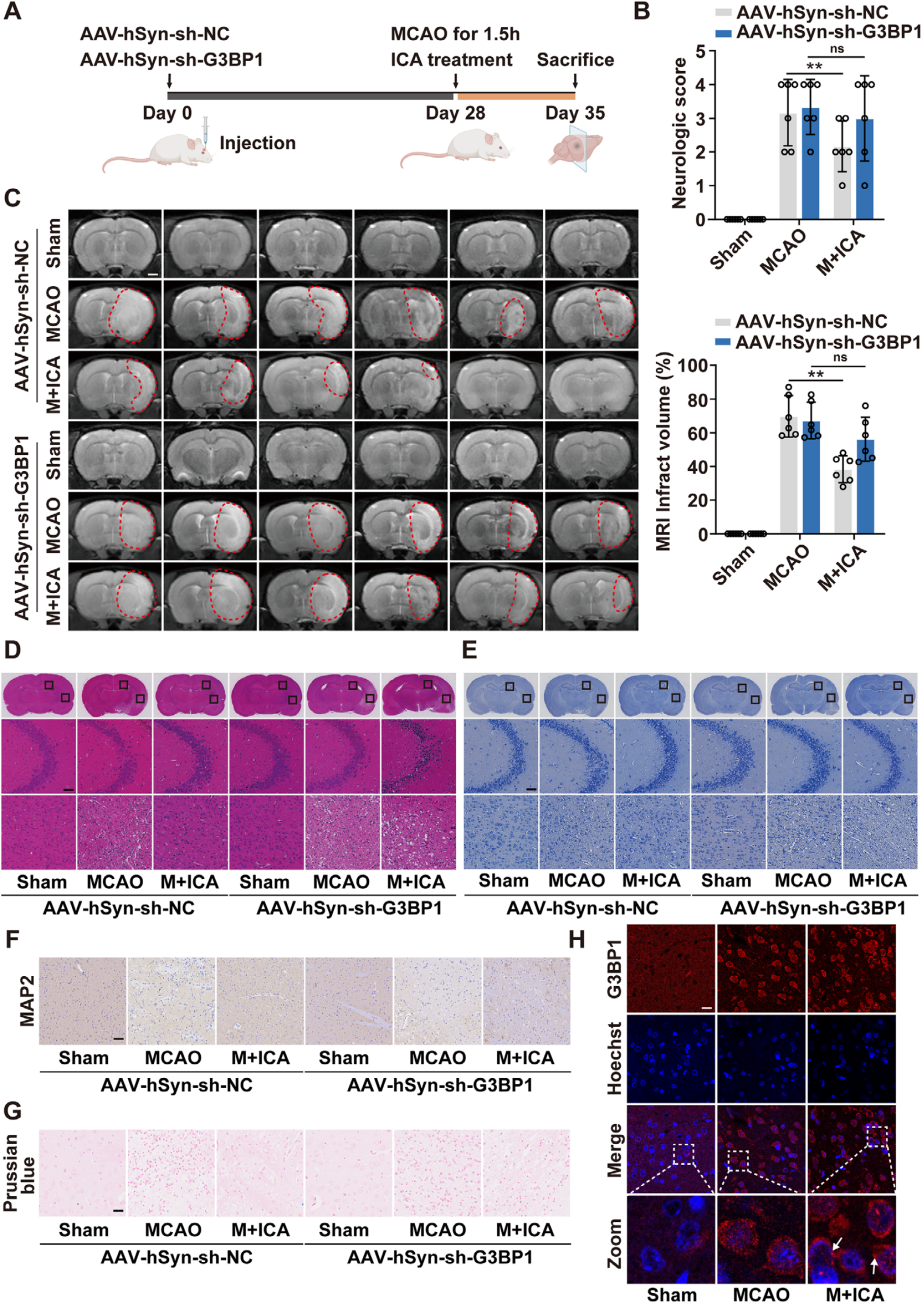
G3BP1 as a therapeutic target in the middle cerebral artery occlusion (MCAO) rat model. A) The evaluation approach for neuroprotection was implemented in the sh‐G3BP1/NC group treated with ICA within the MCAO model. Sham (*n* = 6), MCAO (*n* = 6), and MCAO + ICA (*n* = 6). B) Neurological function score presented the behavioral evaluation of neurological injury in different groups. C) Cerebral infarction volume was measured by MRI and quantified with ImageJ software (bar: 2000 µm). D) H&E staining detected the morphology of cortical and hippocampal tissue (bar: 50 µm). E) Nissl staining presented the morphology of cortical and hippocampal tissue (bar: 50 µm). F) Immunohistochemical analyzed the expression of MAP2 protein in ischemic region (bar: 50 µm). G) Prussian blue staining detected the iron accumulation in infarcted cerebral medulla (bar: 50 µm). H) Immunofluorescence analysis of G3BP1‐associated stress granule aggregation in the ischemic region (bar: 20 µm). Data are expressed as the mean ± SD for 3–6 individual experiments. *p < 0.05, **p < 0.01. ns no significance.

To investigate the role of G3BP1 as a therapeutic target of ICA in ischemic stroke, we employed MCAO model in rat. We performed stereotaxic intracranial injection of specific adeno‐associated‐virus‐9‐hSyn‐small‐short‐hairpin‐RNA‐G3BP1 (AAV‐hSyn‐sh‐G3BP1) into the intracerebroventricular (ICV) of brain to knockdown the expression of G3BP1 in neurons (**Figure** **8**A). The AAV vector was engineered to carry a green fluorescent label, enabling precise anatomical localization through fluorescence microscopy. Robust GFP signal was detected throughout the cerebral cortex following 28 days ICV delivery, confirming successful viral transduction. Furthermore, the expression of G3BP1 significantly decreased compared with the sh‐NC group (Figure S8A,B, Supporting Information).

Subsequently, we constructed the MCAO model and administered ICA via intraperitoneal injection once daily at a dose of 60 mg kg^−1^ for 7 days (M + ICA group). Compared to the sham group, the MCAO group in the sh‐NC exhibited significant neurological deficits, which were attenuated by ICA treatment (Figure 8B). The brain infarct volume was measured by magnetic resonance imaging (MRI), revealing a significantly larger percentage of cerebral infarct area in the MCAO group than the sham group. ICA treatment effectively reduced the percentage of the infarct area (Figure 8C). Additionally, the brain weight of MCAO rats increased compared to sham group, whereas ICA treatment attenuated this effect (Figure S8C, Supporting Information). Moreover, we observed that these therapeutic efficacies of ICA were significantly attenuated in G3BP1 knockdown rats (Figure 8B,C; Figure S8C, Supporting Information). Next, hematoxylin and eosin (H&E) and Nissl staining were used to distinct morphologies in cortex and hippocampal regions. The MCAO group exhibited extensive neuronal damage in these areas, characterized by a loss of normal cellular arrangement, structural damage, widespread pyknotic, hyperchromatic nuclei in pyramidal neurons, and the absence of Nissl bodies. However, ICA treatment markedly ameliorated these pathological morphologies of the cortex and hippocampus regions (Figure 8D,E). Subsequently, we assessed the expression of the neuronal marker MAP2 in cortical neurons by immunohistochemistry (IHC). We found that ICA significantly increased MAP2 levels compared with the OGD/R group in the sh‐NC MCAO group, which was significantly reversed in G3BP1 knockdown rats (Figure 8F). Meanwhile, Prussian blue staining indicated that ICA inhibited ferroptosis in ischemic region (Figure 8G). These protections were attenuated following G3BP1 knockdown (Figure 8D–G). Furthermore, we performed immunofluorescence staining to detect the SGs formation after ICA administration in the ischemic cortex. The results showed that a small amount of SGs appeared in the MCAO group, and this phenomenon was enhanced by ICA treatment (Figure 8H). To assess the systemic safety of ICA, H&E staining of the heart, liver, spleen, lung, and kidney was performed. As shown in Figure S8D (Supporting Information), there was no significant damage observed in these organs in either the sh‐NC or sh‐G3BP1 groups when compared to the sham group. Taken together, these findings indicated that G3BP1 served as a promising therapeutic target for ICA against ischemic cerebral injury.

### 2.9 G3BP1 Represents a Biomarker with Translational Medical Value for Ischemic Stroke

**Figure 9:**
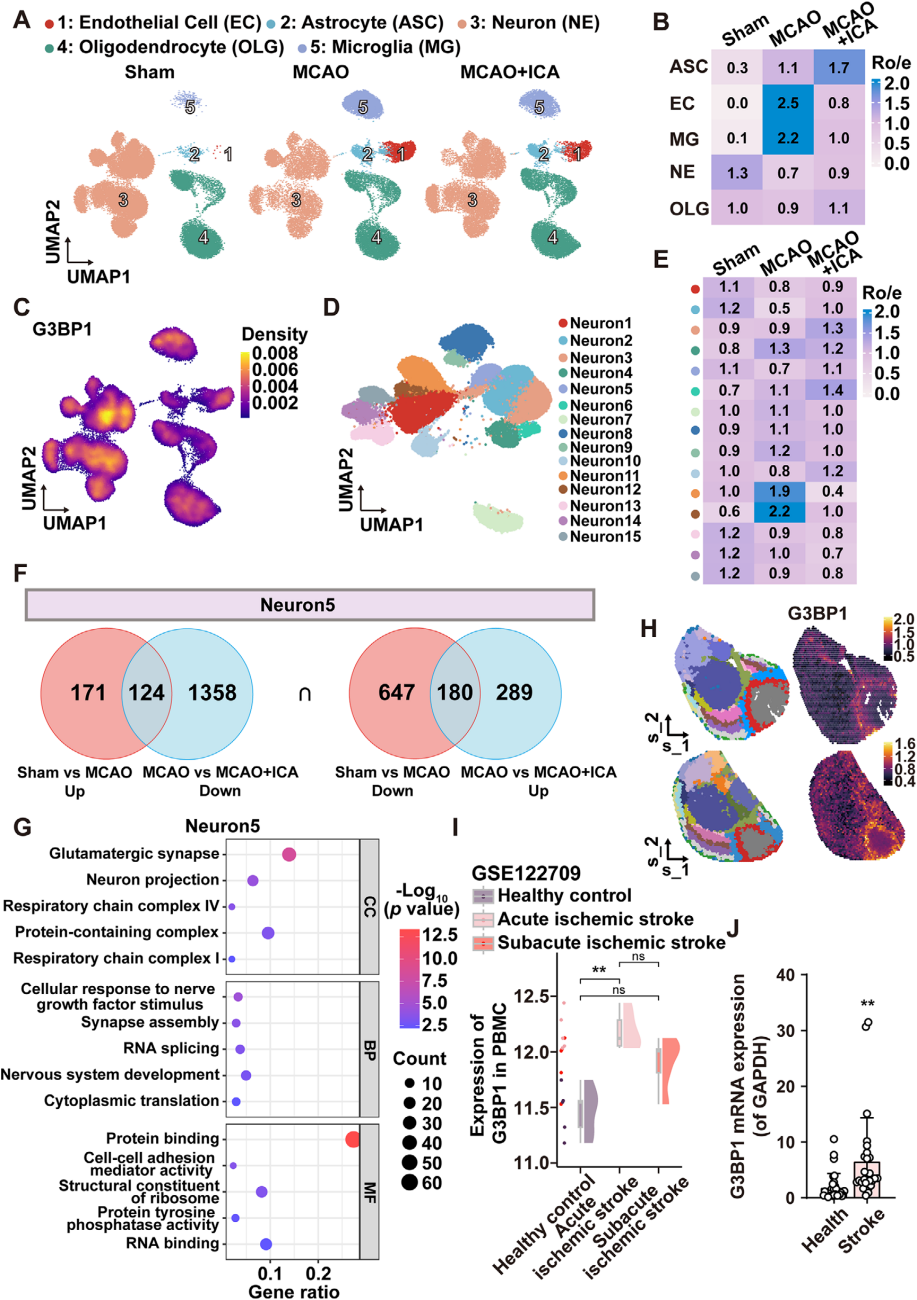
G3BP1 represents a biomarker with translational medical value for ischemic stroke. A) Uniform manifold approximation and projection (UMAP) representation of the snRNA‐seq dataset. Sham (*n* = 3), MCAO (*n* = 3), and MCAO + ICA (*n* = 3). B) Heatmap showed the prevalence of cell cluster, estimated by the Ro/e score. C) Visualization of G3BP1 pertinent to cell cluster. D) UMAP represented the subcluster analysis of the neuron. E) Heatmap showed the prevalence of neuron subcluster, estimated by the Ro/e score. F) Venn diagram presented the modulated DEGs overlapping between Sham versus MCAO up and MCAO versus MCAO + ICA down and Sham versus MCAO down and MCAO versus MCAO + ICA up. G) GO analysis was performed on the DEGs in Neuron 5. The results were sorted using the *p* value and highlighted the top 5 of biological processes (BP), cellular components (CC), and molecular functions (MF). H) Spatial transcriptomics visualization revealed the regional expression patterns of *G3BP1* across distinct brain ischemia center area. The color intensity corresponded to the magnitude of gene expression. I) Expression level of G3BP1 in the peripheral blood sample of healthy controls and stroke patients. J) The mRNA expression of *G3BP1* in blood from patients with and without acute cerebral ischemic stroke (30 patients with acute ischemic stroke and 30 healthy volunteers). Data are expressed as the mean ± SD for at least 3 individual experiments. *p < 0.05, **p < 0.01. ns no significance.

To further validate our findings, we conducted single nucleus RNA sequencing (SnRNA‐Seq) to investigate the ICA related cellular profiles. Brain tissues from the ipsilateral ischemic hemisphere were harvested from three groups: Sham (*n* = 3), MCAO (*n* = 3), and MCAO + ICA (*n* = 3). After stringent quality control, a total of 106 155 were sampled, with 40 628, 31 829, and 33 698 nuclei collected from the Sham, MCAO, and MCAO + ICA groups, respectively. Based on the expression of highly specific marker genes,^[^ ^44^ ^]^ we identified 5 clusters, including Endothelial cell (EC) (*Mgp*, *Col1a1*, *Col1a2*, and *Igfbp2*), Astrocyte (ASC) (*Rorb*, *Itih3*, and *Slc4a4*), Neuron (NE) (*Syt1*, *Slc17a7*, and *Snhg11*), Oligodendrocyte (OLG) (*Mal*, *Plp1*, and *Mbp*), and Microglia (MG) (*Apoe*, *Tgfbr1*, and *Lrmda*) (**Figure** **9**A; Figure S9A,B, Supporting Information). As expected, neuron clusters were depleted derived from severely infarcted tissue, and exhibited neuroprotection after ICA treatment (Figure 9B). Besides, we suspected that ICA treatment could also improve the abnormal activation of microglia and oligodendrocyte rewmodeling in the ischemic area caused by MCAO modeling, from the Ro/e diagram in MG and OLG, which was worth further exploration (Figure S9C–F, Supporting Information). These results suggested that ICA conferred significant protective effect on the survival of neurons in the ischemic region.

Next, we characterized the expression pattern of G3BP1 in different cell types. Notably, G3BP1 transcriptional levels higher in neurons than in the other clusters (Figure 9C). Giving the important role of neurons in nervous system function, we further subclustered analysis of the neuron and identified 15 clusters (Figure 9D). These clusters were classified into excitatory neurons, inhibitory neurons, projection neurons, and special functions neuron according to identified marker genes.^[^ ^44^ ^]^ Excitatory neuron, such as Neurons 1, 2, 3, 4, and 5 were enriched genes including *Rorb*, *Enc1, Epha6*, *Tshz2*, and *Kcnn2*. Moreover, Neurons 13, 14, and 15 were identified as inhibitory neurons, as expressing *Vip*, *Sox6*, and *Sst*. Neuron 5 (*Fezf2*, *Pou3f1*, and *Kcnn2*), Neuron 7 (*Dpp10* and *Tle4*), and Neuron 9 (*Ntng1* and *Tcf7l2*) were considered to be projection neurons. There were also some neurons with special functions, sensory neurons (Neuron 8 with marker genes *Vwc2l* and *Nxph3*) or cerebellar neuron (Neuron 12 with marker genes *Zic1* and *Zic4*) (Figure S9G, Supporting Information). Among the clusters, Neuron 5 highly expressed *G3BP1* genes and the cellular proportion decreased markedly in ischemia brain but obviously recovered after ICA treatment (Figure 9E; Figure S9H,I, Supporting Information). Thus, we performed Gene Ontology (GO) analysis on the DEGs of subpopulation after ICA treatment and identified several GO terms related to neuron projection, cellular response to nerve growth factor stimulus, and protein binding in cellular components (CC), biological processes (BP), and molecular functions (MF) among the top 5 enriched terms (Figure 9F,G).

Moreover, we analyzed data from the Gene Expression Omnibus (GEO) database to explore the relationship between G3BP1 and stroke. Spatial transcriptomics (CRA006645) derived from the mouse MCAO model further demonstrated a pronounced elevation of G3BP1 expression in the proximal region of the periinfarct area compared to the infarct core and the distant periinfarct regions (Figure 9H; Figure S10A, Supporting Information).^[^ ^45^ ^]^ Then, we analyzed G3BP1 expression in peripheral blood mononuclear cells (PBMC) between stroke patients and health control (GSE122709).^[^ ^46^ ^]^ We observed a significant increase in G3BP1 mRNA levels during the acute phase of cerebral ischemia, followed by a decline during the subacute phase. Additionally, with receiver operating characteristic analyze, the area under the curve was 0.940, suggesting that G3BP1 was identified as a valuable biomarker to diagnose this diseased state (Figure 9I; Figure S10B, Supporting Information). To validate the translational relevance of our findings from the in vivo and in vitro models, we further obtained peripheral blood samples from clinical patients with ischemic stroke and healthy subjects. Then, we analyzed the mRNA levels of *G3BP1*, *SCD1*, *PABPC1*, *SLC11A2*, and *ACSL4* in blood from patients with and without acute cerebral ischemic stroke by quantitative real time‐PCR (qRT‐PCR). As shown in Figure 9J and Figure S10C (Supporting Information), the expression of these genes was significantly elevated in the patients with cerebral ischemia, suggesting that these genes were closely related to the development of ischemic stroke. Therefore, the genes such as *G3BP1* may serve as potential biomarkers for assessing the progression of cerebral ischemia, thereby facilitating clinical diagnosis.

## 3 Discussion

G3BP1‐driven SGs assembly is mechanistically linked to disease pathobiology across neurological and metabolic disorders.^[^ ^13^ ^]^ Deciphering the molecular mechanisms that regulate G3BP1 phase separation competence offers a foundational framework for the development of precision modulators targeting biomolecular condensates.^[^ ^10^ ^]^ Thus, this research area holds particularly significant in the context of therapeutics for stroke. In our study, we identify small‐molecule ICA as a chemical inducer of G3BP1 condensation, demonstrating that targeted potentiation of phase separation machinery can achieve neuroprotective efficacy through functional rescue in models of ischemic stroke.

Biochemical studies have established that stress‐inducible assembly of G3BP1‐containing biomolecular condensates constitutes a primordial stress‐adaptation mechanism.^[^ ^13^ ^]^ Although these dynamic granules serve as diagnostic hallmarks of stress‐activated proteostasis pathways,^[^ ^50^ ^]^ prevailing therapeutic paradigms have focused on suppressing condensate formation as a cytoprotective intervention. For example, inhibiting stress granule formation can prevent the aberrant folding and aggregation of TDP‐43 and tau proteins, thereby mitigating the malignant progression of neurodegenerative diseases.^[^ ^47^ ^]^ Simultaneously, while SGs function as a spontaneous protective mechanism for cells against damage, it is probable that enhancing the formation of SGs can effectively maintain RNA stability and, consequently, safeguard against cellular damage under stress conditions.^[^ ^48^ ^]^ Of note, recent investigations have emphasized the beneficial role of SGs assembly in enhancing cellular self‐preservation by regulating gene expression, revealing therapeutic potential across various human diseases.^[^ ^35^, ^49^ ^]^ Additionally, several compounds, including TC6, TC12, TC39, and TC41, have been identified as SGs inducers through binding to the NTF2L domain of the G3BP1 via an in silico structure‐based approach. However, the potential cytoprotective and therapeutic applicability remain largely unexplored in human disease contexts.^[^ ^21^, ^50^ ^]^ Therefore, our study represents the first exploration of small‐molecule inducing cells to produce controllable levels of SGs, offering a promising avenue for future disease treatment and drug development.

Notably, various environmental stimuli such as nutrient deprivation, heat shock, or oxidative stress can induce the formation of SGs in cells.^[^ ^51^ ^]^ However, this process may lead to uncontrolled SGs accumulation.^[^ ^49^ ^]^ The formation of SGs depletes substantial quantities of intracellular substances and energy, compromising cellular self‐protective functions and exacerbating existing damage.^[^ ^52^ ^]^ Therefore, the prerequisite for cellular protection through the promotion of SGs formation is to maintain SGs level within a controllable range.^[^ ^3^ ^]^ Our results indicate that the efficacy of ICA in promoting the formation of SGs is inferior to that of arsenite. Therefore, ICA dose not induce the excessive formation of SGs, as observed with arsenite. This discrepancy may also account for the differences in the cellular biological phenotypes elicited by ICA and arsenite. In the future, it may be possible to develop a class of compounds that can induce the formation of SGs through moderate stimulation. These compounds may facilitate the controlled induction of SGs formation in cells, thereby providing cytoprotective potentials and therapeutic effects for various human diseases.

Molecular binding assays reveal a direct interaction between ICA and the NTF2L domain of G3BP1. Meanwhile, ICA covalently modifies K50, which resides within residues 37–59, a pocket located helix αIII and sheet βI. Thus, we propose a dual‐binding mechanism for the interaction between ICA and G3BP1, predominantly mediated by hydrogen bonds and covalent modifications. Molecular docking suggests that ICA initially docks into the Lys50‐proximal binding pocket stabilized by hydrogen‐bonding networks, subsequently inducing conformational rearrangements that position the Lys50 residue in proximity to the α, β‐unsaturated double bond, thereby facilitating covalent adduct formation. Therefore, this region provides a novel binding site for future drug design, distinct from previously reported inhibitors or activators of the G3BP1 protein, such as Caprin‐1 or USP10.^[^ ^17^, ^53^, ^54^ ^]^

Moreover, our research has revealed that ICA directly binds to the NTF2L domain of G3BP1, thereby promoting the phase separation, which has not been reported previously. We observe that ICA accelerates fluorescence recovery during G3BP1 oligomerization. This phenomenon may be attributed to several factors, including physical properties of the phase environment, phase‐separation‐driven active transport, biomolecular surfactants, intermolecular interaction dynamics, and the experimental system complexity. Thus, this potentially explains the observed FRAP characteristics in our study. In particular, ICA binds to the groove formed by the two NTF2L domains in G3BP1 dimer, which serves as an ideal drug‐binding pocket. Of note, within this chemical microenvironment, ICA interacts with surrounding amino acids and causes allosteric regulation through various intermolecular interactions. Here, we speculate that ICA may induce the proximity of the two NTF2L domains in a molecular glue‐like manner, thereby accelerating G3BP1 dimer formation and ultimately promoting phase separation. Our findings indicate that ICA likely binds to the Lys50 proximal binding pocket, stabilizing the dimeric structure and promoting oligomerization through hydrogen bonding and covalent interaction. Thus, ICA confers neuroprotection against cerebral ischemia‐reperfusion injury through the modulation of downstream signaling pathways targeting G3BP1. Consequently, these results establish a foundational basis for designing small‐molecules that specifically target the NTF2L domain, enabling the development of molecular probes to promote G3BP1 oligomerization and further facilitate the exploration of the phase separation mechanism. Additionally, our findings present a promising molecular framework for developing potential therapeutics targeting G3BP1 phase separation (Figure S10D, Supporting Information).

Our study establishes targeted activation of G3BP1‐mediated stress granule assembly as a mechanism‐driven cytoprotective strategy, unveiling pharmacological potentiation of biomolecular condensation as a druggable pathway for ischemic cerebral protection. These findings provide mechanistic validation for harnessing phase separation dynamics in neuroprotective therapy development.

## 4 Experimental Section

### Chemicals and Reagents

ICA (C_33_H_40_O_15_, molecular mass: 676.24, Cat#T2855) was purchased from TargetMol (Boston, MA, USA). The purity exceeded 98%.

### Cell Lines

The PC12 (Cat# TCR9, RRID: CVCL_0481) and HEK293T (Cat# SCSP‐502, RRID: CVCL_0063) cells were purchased from Cell Bank of Shanghai Institute of Biochemistry and Cell Biology, Chinese Academy of Sciences (Shanghai, China). Cells were cultured in Dulbecco's modified Eagle's medium (DMEM) supplemented with 1% penicillin–streptomycin and 10% fetal bovine serum (Gibco, Cat# 10270106), then maintained at 37 °C with 95% air and 5% CO_2_ under a humidified incubator. The U2OS cells stably overexpressing G3BP1–GFP was kindly provided by the Pei‐Pei Zhang Laboratory (Peking university, Beijing, China). This cell line was originally obtained from Cell Bank of Shanghai Institute of Biochemistry and Cell Biology (Cat# SCSP‐5030, RRID: CVCL_0042) and cultured as described above.

Primary cortical neurons were obtained from the cortical tissue of 18 days embryonic Sprague‐Dawley rats. Briefly, the cerebral cortices were isolated and digested with 0.25% trypsin at 37 °C for 30 min, after which dissociated cells were filtered with 40 µm cell strainer and maintained in neurobasal medium supplemented with 1% GlutaMAX (Gibco, Cat# 35050061), 2% B‐27 supplement (Gibco, Cat# 17504‐044), and 1% penicillin–streptomycin (Gibco, Cat# 15140‐122). Cells were plated at 5.0 × 10^4^ cells cm^−2^ on poly‐l‐lysine (Sangon Biotech, Cat# A600751) precoated plates and used for experiments at 14–21 days in vitro.

### Recombinant Protein Expression and Purification

The full‐length human G3BP1 or its mutant variants were cloned into pGEX‐4T1 vector which contained a thrombin cleavage site between the N‐terminal GST and fusion protein. The recombinant protein was expressed and purified from *Escherichia coli* BL‐21 (DE3). Briefly, *E. coli* was grown in medium until optical density (OD) 600 = 0.8, followed by induction with 0.5 mm isopropylthio‐β‐galactoside (Solarbio, Cat# I8070) for 12 h, 200 rpm at 16 °C. After harvesting, cells were sonicated with lysis buffer (250 mm NaCl, 50 mm HEPES, 1 mm DTT, pH 7.5, and protease inhibitor). The supernatants were applied to a GST column (Cytiva, Cat# 17075605) and then eluted with 10 mm glutathione (Sigma, Cat# V900456). The elution was further incubated with thrombin overnight at 4 °C and loaded on equilibrated Superdex 200 increase 10/300 GL (Cytiva, Cat# 28990944) and HiTrap Q column (Cytiva, Cat# 29051325) to remove GST and thrombin. The fractions were flash‐frozen and stored at −80 °C until use.

### Cell Transfection

For G3BP1 and its mutant variant overexpressions, G3BP1, G3BP1^1–139^, G3BP1^1–334^, and G3BP1^335–466^ cDNA with different tags were cloned into overexpressing vector pcDNA3.1 or pEGFP‐N1 vectors. HEK293T cells were transfected with polyethyleneimine and PC12 cells were transfected with lipofectamine 2000 (Invitrogen, Cat# 11668500). Cells were used for subsequent experiments after transfecting with plasmids at least for 24 h.

sh‐G3BP1 (5′‐CAAAUUCUAUGUUCAC AAUTT‐3′, 3′‐AUUGUGAACA UAGAAUUUGTT‐5′), si‐IGF2BP1 (5′‐CUUUCUCGGGGAAAGUAGAAU‐3′, 3′‐UCUACUUUCCCCGA GAAAGUU‐5′), and their control were purchased from Hanbio Biotechnology (Shanghai, China). PDE5‐specific short interfering RNA (si‐PDE5, 5′‐GGACAGCUCUAAAGACAAAT T’, 3′‐UUUGUCUUUAGAGCUGCCTT‐5′) and its control were purchased from Genomeditech (Shanghai, China). Cells were infected with sh‐G3BP1/NC lentivirus, followed by addition of 5 µg mL^−1^ of puromycin (Invitrogen, Cat# A1113803), to establish a stable knockdown cell line. si‐IGF2BP1, si‐PDE5, or si‐NC was transfected with Lipofectamine RNAiMAX (Invitrogen, Cat# 13778150).

### High Content Screening

A compound library consisted of 945 compounds was purchased from TargetMol (Boston, MA, USA). Briefly, after transfection with GFP–G3BP1 plasmids, the cells were treated with compounds at a final concentration of 10 µm for 24 h. The fluorescence puncta in cells were acquired and counted using an Operetta High‐Content imaging system (PerkinElmer, Waltham, MA, USA). Compounds that induced puncta more than 2.5‐fold were selected as final hits.

### LLPS Analysis

For in vitro LLPS experiments, the purified 100 µm recombinant G3BP1 and GFP–G3BP1 proteins were incubated with different concentrations of ICA (5, 10, and 20 µm) prior to RNA induction (100 ng µL^−1^) and 10% bovine serum albumin (BSA) as crowding agent in buffer. The protein–RNA mixture was applied onto a 96‐well glass bottom plate and visualized using DIC or fluorescence imaging with LSM880 confocal microscope (Zeiss, Oberkochen, Germany). For in vivo LLPS experiments, cells were transfected with plasmids and treated with 5, 10, and 20 µm ICA in various times followed by imaging on LSM880 confocal microscope (Zeiss).

### FRAP Analysis

FRAP experiments were performed on Nikon A1 confocal microscope (Nikon, Tokyo, Japan) with 100 × /1.4 NA oil immersion objective and 200 mW 488 nm laser line. The circular regions, selected from G3BP1 puncta (in vivo) or G3BP1 condensates (in vitro), were bleached once using 50% laser power for 2 s. Fluorescence recovery was recorded over 30 s at 2 s intervals. The fluorescence intensity after bleaching was normalized to the intensity of the prebleaching. FRAP data were analyzed using NIS‐Elements Viewer 5.21.00.

### Western Blotting

The cells were lysed with NP‐40 buffer. Then, the resulting supernatants were mixed with loading buffer and subjected to sodium dodecyl sulfate–polyacrylamide gel electrophoresis (SDS‐PAGE) separation. The proteins were then transferred onto polyvinylidene fluoride (Millipore, Cat# IPVH00010) membrane and blocked with 5% BSA. The membrane was further incubated with PKR (Abcam, Cat# ab184257), PERK (Abcam, Cat# ab229912), eIF2α (CST, Cat# 9722), p‐eIF2α (CST, Cat# 3398), G3BP1 (Proteintech, Cat# 66486‐1‐Ig;), G3BP2 (CST, Cat# 31799), IGF2BP1 (CST, Cat# 8482S), P70S6K (CST, Cat# 9202), p‐P70S6K (CST, Cat# 9234), GPX4 (Biodragon, Cat# BD‐PN3047), AMPK (CST, Cat# 5831), p‐AMPK (CST, Cat# 2535), mTOR (CST, Cat# 2972), p‐mTOR (CST, Cat#2971), p38 (CST, Cat# 9212), p‐p38 (CST, Cat# 9211), ERK (CST, Cat# 4695), p‐ERK (CST, Cat# 4370), and GAPDH (Proteintech, Cat# 60004) antibodies overnight, followed by incubation with the corresponding HRP‐linked secondary antibody. The proteins signals were detected with Tanon 5200 multi‐chemiluminescent imaging system (Tanon, Shanghai, China).

### SPR Analysis

Biacore T200 system (GE Healthcare) was used for SPR analysis. Briefly, recombinant G3BP1 and G3BP1 variants (G3BP1^1–139^/G3BP1^1–334^/G3BP1^335–466^) were coupled onto CM5 sensor chip (Cytiva, Cat# BR100399) with acetic acid (pH 4.5). For screening, compounds were detected at a final concentration of 50 µm. For kinetic analysis, compounds were diluted in various concentrations from 0.098 to 50 µm. Time parameters of combination and dissociation were set as 60 and 120 s, respectively. Data were analyzed with Biacore evaluation software (T200 v2.0). *K*_D_ values were calculated by fitting kinetic data using the 1:1 Langmuir binding model or affinity.

### ITC Analysis

ITC experiment was performed using a MicroCal PEAQ ITC system (Malvern, Worcestershire, UK). In brief, ICA (20 µm) and recombinant G3BP1 (140 µm) protein was dissolved in phosphate‐buffered saline (PBS) containing 5% DMSO. Before measurements, both solutions were degassed. The sample cell and injection syringe were cleaned with Decon‐90, water, and methanol. A total of 19 titrations were performed with 150 s intervals between injections. The initial single titration was 0.4 µL over 0.8 s, followed by 18 titrations with 2 µL injections over 4 s. The experiment was conducted at 25 °C with a stirring speed of 125 rpm. The background correction was achieved by titrating protein into PBS. Data analysis was executed using Nano Analyzer 2.1 software.

### MST Analysis

For recombinant protein MST detection, the protein was labeled with a red‐NHS labeling kit (NanoTemper). The recombinant G3BP1 protein was incubated with various concentrations ICA at 4 °C for 1 h. For cell lysis MST detection, the HEK293T cells were transfected with GFP‐fused G3BP1 and its mutants G3BP1^1–139^/G3BP1^1–334^/G3BP1^335–466^, and GFP–plasmid used as control. Cells were freeze–thawed multiple times with liquid nitrogen. The supernatant of cell lysate was used to detect and ICA treatment was the same as above. The mixture was loaded into standard capillaries and binding affinity was examined using a Monolith NT.115 instrument (NanoTemper Technologies, Munich, Germany). Data were analyzed using MO affinity analysis software (NanoTemper Technologies).

### DARTS Assay and MS Analysis

For DARTS in cell, cells were incubated with 5, 10, and 20 µm ICA or DMSO for 4 h. After that, 5 µg mL^−1^ pronase (Roche, Cat# 10165921001) was added for 20 min at room temperature. The expression of G3BP1 was detected with western blot. For DARTS–MS analyze, the recombinant G3BP1 protein was incubated without or with ICA (20 µm) at 4 °C for 1 h, followed by addition of pronase. The mixture was separated by SDS‐PAGE gel with Coomassie bright blue staining. The trypsin‐digested gel bands were analyzed in the same way as LC–MS/MS analysis.

### CETSA

Cells were treated with 20 µm ICA or DMSO for 4 h, and then heated for 3 min at a specified temperature ranging from 37 to 64 °C. Moreover, cells were collected and freeze–thawed 5 times with liquid nitrogen in kinase buffer. The expression of G3BP1 was detected with western blot.

### Pull‐Down Assay

ICA–Fe_3_O_4_ nanoparticles were synthesized as previously reported.^[^ ^28^ ^]^ Briefly, ICA was linked to the DHBP‐bound Fe_3_O_4_ NPs via UV radiation. ICA‐NPs were incubated with cell lysates in the presence or absence of ICA (40 µm) for competitive binding overnight at 4 °C. Then, the beads were washed with PBS containing 0.1% triton X‐100. The beads‐captured proteins were obtained and separated through SDS‐PAGE for further western blot using G3BP1 antibody.

### LC–MS/MS Analysis

The recombinant G3BP1^1–139^ protein was incubated without or with ICA at 4 °C overnight. The mixture was separated using SDS‐PAGE, followed by the excision of the bands and subsequent trypsin digestion. The peptides were analyzed by liquid chromatography coupled with LTQ‐Orbitrap velos pro mass spectrometer (Thermo Fisher Scientific, MA, USA), equipped with a nanoelectrospray ion source, an analytical column (75 µm, 10 cm), and 3 µm reversed phase (RP) C18. Elution was performed with the following gradient: 2–40% B for 70 min; 40–95% B for 5 min; 95% B for 20 min (solvent A: 0.1% formic acid in water, solvent B: 0.1% formic acid in acetonitrile). Full scan MS spectra were acquired from *m*/*z* 350–2000 with a resolution of 60 000 (FWHM) in the Orbitrap analyzer. The top 15 most abundant precursor ions with charge states at least 2 were selected for MS/MS scans. Mass spectrometric data were processed using Proteome Discoverer software with the SEQUEST search engine with the following parameters: methionine oxidation (+15.9950 Da), cysteine carbamidomethylation (+57.0210 Da), lysine in G3BP1^1–139^ (+676.2400 Da), precursor mass tolerance of 10 ppm, and a fragment ion mass tolerance of ±0.8 Da. The false discovery rate (FDR) threshold was of 0.01.

### G3BP1 Multimer and Protein Cross‐Linking Mass Spectrometry (XL–MS) Analysis

The XL–MS experiment was carried out according to previous methods.^[^ ^55^ ^]^ ICA was incubated with recombinant G3BP1^1–139^ at 4 °C for 1 h. Chemical cross‐linker DSS (2 mm) (Sigma, Cat# S1885) was then added to the mixture, followed by incubation at RT for 30 min. The reaction was terminated by 100 mm Tris‐HCl. Subsequently, the sample isolation and peptide analysis were performed as described above. The cross‐linked peptide pairs were identified by pLink 2 software^[^ ^56^ ^]^ with the following criteria: cross‐linking sites, Lys; cross‐linker, DSS; cross‐link (+138.0680 Da); monolink (+156.0786 Da); peptide length, 4–100 amino acids per chain; peptide mass, 400–10 000 Da; precursor mass tolerance of 20 ppm; fragment mass tolerance of 20 ppm; and FDR threshold of 0.01.

### BiFC Analysis

GFP (1–155)–G3BP1 and GFP (156–238)–G3BP1 BiFC constructs were subcloned in the expression vector pcDNA3.1.^[^ ^29^ ^]^ Cells were cotransfected with plasmids and subsequently treated with ICA for 24 h. The fluorescence images were captured through the LSM880 confocal microscope (Zeiss).

### DLS Assay

DLS experiments were conducted using a Zetasizer Nano ZS instrument (Malvern Instruments, Malvern, UK), employing 10 mm square polystyrene cuvettes (Malvern Instruments). Sample solutions contained 10 µm G3BP1, either alone or combined with various concentration of ICA with or without RNA and PEG. Prior to analysis, all solutions were filtered through 0.22 µm membranes. All experiments were carried out at room temperature, with each condition measured using 10 scans per run. Data were processed using the Malvern Zetasizer Software.

### Tryptophan Fluorescence Quenching Assay

Briefly, recombinant G3BP1 protein was incubated with various concentrations of ICA (0–25 µm). Tryptophan fluorescence quenching was monitored by excitation wavelength at 370 nm and emission wavelengths from 300 to 500 nm with a slit width of 1 nm using a fluorescence spectrophotometer (PerkinElmer). Fluorescence intensity was corrected by the blank solution emission spectra.

### CD Analysis

CD analysis was performed with a JASCO J‐810 circular dichroism spectropolarimeter (JASCO, Tokyo, Japan). Briefly, recombinant G3BP1 protein was incubated with various concentrations of ICA (0–50 µm) at room temperature. CD measurements were conducted under ambient conditions with a scan rate of 100 nm min^−1^ and a response time of 1 s. Quartz cuvettes with a 0.1 cm pathlength were employed for analysis within the wavelength range of 180–280 nm.

### HDX‐MS Analysis

As previously described,^[^ ^32^ ^]^ G3BP1 protein was incubated with or without ICA (50 µm) at 4 °C for 30 min. The deuterium labeling reaction of protein was equilibrated in D_2_O buffer (containing 100 mm phosphate, pD 7.00) at room temperature and quenched with 37.5% hydrochloric acid at indicated times (0.167, 0.5, 1, 5, 10, 30, 60, and 120 min). Samples underwent an online digestion process through Waters Enzymate BEH pepsin column (2.1 × 30 mm, 5 µm). Then, the resulting peptides were directly captured and eluted from the VanGuard Pre‐Column trap with 15% acetonitrile. Subsequently, these peptides were separated on an Acquity UPLC BEH C18 column. Mass spectrometric data were acquired using a Waters Xevo G2 mass spectrometer. Peptide identification was performed using ProteinLynx Global Server 3.0.2. Relative deuterium levels of all peptides were determined by comparing the mass of deuterium‐labeled peptides to nondeuterated controls. Data processing was conducted using DynamX 3.0 (Waters corporation, Milford, MA, USA).

### MD Simulations

The crystal structure of human G3BP1 NTF2L domain (PDB: 8V1l) was downloaded from the Protein Data Bank. Molecular docking was processed using the Schrödinger 2018 software. MD simulations were carried out with the AMBER 22 package, employing the PMEMD engine. The equilibrium phase involved a 200 ps simulation performed at increasing temperatures from 0 to 300 K under a weak force field (5 kcal mol^−1^ Å^−^^2^) applied to the complex. Finally, a 500 ns production simulation was conducted under constant temperature (300 K) and pressure (1 atm).

### Active Dimethyl Labeling Analysis

The active dimethyl labeling of lysine in G3BP1 was performed according to an established methodology.^[^ ^33^ ^]^ Briefly, the purified human G3BP1 (1 mg mL^−1^) was incubated with (heavy) or without (light) ICA for 30 min. Samples were isotopic labeled for the 5 min with light solution (30 mm CH_2_O, 5 mm NaBH_3_CN) or heavy solution (30 mm CD_2_O, 5 mm NaBH_3_CN). The reaction was quenched with NH_4_HCO_3_, and the buffer was exchanged to 20 mm NH_4_HCO_3_ by centrifugal filters. Samples were further incubated with 6 m urea and 20 mm DTT for 2 h, and then 40 mm IAA was added for 40 min in the dark. Salts and urea were removed by centrifugal filters. The mixture was digested with thermolysin at 65 °C for 5 h and the two portions were pooled. The labeled peptides were quantitated by LC–MS/MS analysis as above with the following criteria: lysines with light label (+28.0313 Da); lysines with heavy label (+34.0631 Da).

### NMR Spectroscopy

The ^15^N‐labeled G3BP1^1–139^ was produced by culturing and inducing the DE3 in M9 minimal media supplemented with ^15^NH_4_Cl (Merck, Cat# 299251). The labeled protein was dissolved in 50 mm HEPES, 50 mm NaCl, 2 mm DTT, and 10% D_2_O at pH 7.5. The molar ratio of G3BP1^1–139^ to ICA, respectively, was 1:0, 1:1, 1:2, and 1:4. The samples were added into a NMR tube with an outer diameter of 5 mm. 2D ^1^H–^15^N heteronuclear single quantum coherence spectra were acquired on a 600 MHz spectrometer (Bruker, MA, USA). Data were processed by Topspin and CcpNmr software.

### SILAC‐Based Proteomic Analysis

HEK293T cells were cultured in DMEM medium, while the l‐lysine and l‐arginine were replaced by ^12^C_6_^14^N_4_ l‐arginine and ^12^C_6_^14^N_2_ l‐lysine (light) or ^13^C_6_^15^N_4_ l‐arginine (Thermo, Cat# 88434) and ^13^C_6_^15^N_2_ l‐lysine (Thermo, Cat# 88209) (heavy), until achieving 95% incorporation. The labeled cells were transfected with the HA–G3BP1 plasmids and then treated with ICA (heavy) or DMSO (light). After lysis, the supernatant was incubated with mouse anti‐HA magnetic beads (Invitrogen Cat#88836) ornon‐specific mouse IgG antibody as a control at 4 °C overnight. Proteins were separated by SDS‐PAGE and identified by LC–MS/MS. The STRING database (v12.0) was used to construct PPI associated with the increased interactions of the G3BP1 protein. Data visualization was performed using Cystoscape software (v3.9.1).

### RNA‐Seq Analysis

The RNA sequencing was performed by Novogene Bioinformatics Technology (Beijing, China). Briefly, mRNA was purified from total RNA using oligo(dT) magnetic beads. Subsequently, the first and second strands of cDNA were synthesized based on mRNA fragments as templates. After purification using the AMPure XP system and subsequent quality control, the cDNA libraries were clustered on a cBot Cluster Generation System with the TruSeq PE Cluster Kit v3‐cBot‐HS (Illumina) and sequenced on an Illumina NovaSeq platform. Differential expression analysis was performed using the DESeq2 package (v1.20.0). Differential expression analysis followed by GO and KEGG enrichment was conducted using the clusterProfiler package.

### m^6^A Dot Blot

The m^6^A dot blot assays were conducted to assess the overall abundance of m^6^A. Briefly, RNA samples were combined with incubation buffer and heated at 65 °C for 5 min, followed by dilution in saline‐sodium citrate buffer. Samples were blotted onto the Hybond‐N+ membrane (Biotopped, Cat# RPN303B) using the Bio‐Dot Apparatus and subjected to UV irradiation at 254 nm for 5 min. The samples were incubated with rabbit anti‐m^6^A antibody (Proteintech, Cat# 68055‐1‐Ig). The identical membrane was stained with methylene blue to serve as a loading control.

### MeRIP‐Seq and MeRIP‐qPCR

Cells with OGD/R or OGD/R + ICA were collected. MeRIP‐seq was performed by CloudSeq Biotech (Shanghai, China). In brief, the total RNA immunoprecipitation was carried out using the GenSeq m^6^A‐Methylation kit (GenSeq, Shanghai, China). The RNA was fragmented to an approximate size of 200 nt using RNA fragmentation reagents. The m^6^A antibody–protein A/G beads complexes were subsequently mixed with RNA fragments and incubated at 4 °C for 4 h. The captured RNA was eluted and purified. Libraries for both IP and input samples were prepared using the GenSeq Low Input Whole RNA Library Prep Kit (GenSeq, Shanghai, China). The library quality was assessed with an Agilent 2100 Bioanalyzer (Agilent Technologies, Palo Alto, CA, USA), and the libraries were sequenced on the NovaSeq platform (Illumina, San Diego, CA, USA) via paired‐end sequencing under Q30 read quality standards. The 3′ adapter sequences and low‐quality reads were trimmed using cutadapt software (v1.9.3). Methylated RNA sites (peaks) were identified using MACS software. The diffReps software was used to detect differentially methylated sites with a fold‐change cutoff of 2 and a *p*‐value < 0.00001. The RIP kit was used to perform MeRIP‐qPCR. Briefly, cells were lysed with lysis buffer, and a portion of the lysate was retained as input. The m^6^A antibody or IgG, along with protein A/G beads, were added and incubated at 4 °C for 12 h. The beads were washed with wash buffer, then treated with protein kinase K buffer and incubated with elution buffer at 55 °C for 30 min. RT‐qPCR was performed using the reverse transcription kit and the polymerase chain reaction kit. The primer sequences are provided in Table S1 (Supporting Information).

### OGD/R

Cells were cultured without oxygen and glucose to simulate ischemic injury in vitro. Briefly, after removing the supernatant, cells were cultivated with Earle's balanced salt solution in a hypoxia incubator (CCL‐050B‐8, Esco, Changi, Singapore) with 1% O_2_, and 5% CO_2_ for 4 h. Following reoxygenation, the medium was replaced with a complete culture medium containing 5, 10, and 20 µm ICA to facilitate cell culture for 24 h. Cells were maintained under normoxic conditions as a control.

### Cell Viability Assay

PC12 cells and primary cultured rat cortical neurons were seeded, respectively, in 96‐well plates. ICA (5, 10, and 20 µm) was treated following OGD/R. Subsequently, the cells were incubated in the dark with the MTT working solution for 4 h at 37 °C. The formazan product was dissolved in DMSO and measured at 490 nm by Bio‐Rad microplate reader (Bio‐Rad, Hercules, CA, USA).

### AO/EB Staining

AO/EB staining was executed to identify apoptosis. Briefly, cells were treated with the AO/EB solution (Coolaber, Cat# SL7650) for 20 min at 37 °C and imaged through Olympus IX73 fluorescence microscope (Olympus, Tokyo, Japan) under excitation/emission wavelengths of 490/530 nm for AO and 520/590 nm for EB.

### Crystal Violet Staining

For crystal violet staining, cells were fixed with 4% paraformaldehyde and then stained with 0.1% crystal violet solution (Beyotime, Cat# C0121) followed by imaging through Olympus IX73 fluorescence microscope.

### ROS Analysis

To detect intracellular hydrogen peroxide and mitochondrial superoxide anion levels, cells were stained with DCFH‐DA (10 µm) (Invitrogen, Cat# C400) and MitoSOX Red (20 µm) (Invitrogen, Cat# M36008) for 30 min at 37 °C. Images were captured through Olympus IX73 fluorescence microscope.

### Zebrafish Swimming Performance Analysis

Zebrafish were maintained at 28  ±  1 °C with 14 h light/10 h dark cycle container as previously described.^[^ ^57^ ^]^ For hypoxia experiments, zebrafish were maintained in a 0.5 mg L^−1^ low‐concentration oxygen environment for 5 min to simulate acute cerebral hypoxia injury. After treatment, zebrafish was transferred to a 48‐well plate (1 fish per well), and the swimming behavior along with total distance was recorded for 10 min through a video tracking system (Viewpoint ZebraLab, Lyon, France). All zebrafish experiments were approved by the Animal Ethics Committee of Shandong First Medical University (202505).

### Neuron‐Specific G3BP1 Knockdown Viral Constructs

Short hairpin RNA (shRNA) targeting G3BP1 was constructed and delivered into an adeno‐associated virus 2/9 with the neuron‐specific synapsin 1 (hSyn) promoter (Hanbio, Shanghai, China). The sequences were as follows: 5′‐CAAAUUCUAUGUUCACAAUTT‐3′, 3′‐AUUGUGAACAUAGAAUUUGTT‐5′. AAV9‐shRNA‐NC/G3BP1 were packaged with pAAV‐RC and pHelper and used the triple‐plasmid transient transfection method (HB infusion Kit, Hanbio). After purification, viral particles were utilized in subsequent experiments.

### Rat MCAO Model

Male Sprague‐Dawley rats (250–280 g) were sourced from the Animal Center of Peking University Health Science Center. All animal procedures received approval from the Ethics Committee of Experimental Animal Ethics Committee of Peking University (DLASBD0350). AAV vectors encoding sh‐NC or sh‐G3BP1 were administered via ICV injection using a micro syringe (Hamilton, NV, USA) 28 days prior to the experiment. The stereotaxic coordinates for injection were −1 mm anterior/posterior, 2 mm medial/lateral, and −3.8 mm dorsal/ventral relative to bregma. Knockdown efficiency was confirmed by fluorescence imaging and western blot. The sh‐G3BP1 and NC groups were further subdivided into sham, MCAO, and MCAO + ICA subgroups (*n* = 6 per group). The model of MCAO was established by ligating the right external carotid artery (ECA) was ligated and inserting a round‐tipped silicone nylon thread (RWD Life Science, Guangzhou, China) through the stump of the ECA into the right common carotid artery, advancing gently to occlude the right middle cerebral artery at a depth of 18 to 20 mm. The artery was occluded for 1.5 h, after which the thread was removed to allow reperfusion. ICA was administered via intraperitoneal injection once daily at a dose of 60 mg kg^−1^ for 7 days.

### MRI

After the final drug administration, the rat's head was immobilized and positioned in a 40 mm experimental coil for imaging. MRI scanning was performed using a 3.0 T GE 750 superconducting MRI scanner (GE Healthcare). T2‐weighted images were acquired with a fast spin‐echo sequence under the following parameters: repetition time, 3767 ms; echo time, 55.3 ms; field of view, 4 cm; matrix, 129 × 256; number of excitations, 1; number of slices, 11; slice thickness, 1.5 mm; slice gap, 0 mm. Image analysis was conducted using Image software, and the infarct area was calculated on the maximal coronal slice.

### Neurological Function Score

Neurological function in rats was evaluated according to the modified Longa scoring system.^[^ ^58^ ^]^ The scoring criteria were as follows: 0, normal with no deficit; 1, mild deficit (left forepaw unable to fully extend); 2, moderate deficit (left turning); 3, severe deficit (left tilting); 4, no spontaneous movement with reduced consciousness; 5, death. Rats scoring between 1 and 3 points at 24 h postsurgery were included for further analysis.

### Nissl Staining

Brain tissues were fixed in 4% paraformaldehyde, embedded in paraffin, and sectioned into 5 µm thick slices. The sections were deparaffinized and rehydrated, followed by Nissl staining. After staining, the slices were imaged using a digital pathology slide scanner (NanoZoomer, Hamamatsu Photonics, Hamamatsu, Japan).

### H&E Staining

The fixed brain, spleen, liver, heart, lung, and kidney tissues slices were subjected to H&E staining and imaged using a digital pathology slide scanner (NanoZoomer).

### Prussian Blue Staining

Brain sections were incubated in Prussian blue staining solution for 20 min.^[^ ^59^ ^]^ Afterward, the sections were counterstained with nuclear fast red, dehydrated, and cleared in xylene. Finally, the sections were mounted with a resinous medium and coverslipped. Images were captured with digital pathology scanner (NanoZoomer).

### IHC and IF Detection

For IHC assay, brain tissues were fixed in 4% paraformaldehyde, embedded in paraffin, and sectioned into 5 µm thick slices. Endogenous peroxidase activity was quenched with 3% methanol–H_2_O_2_ solution, followed by blocking nonspecific binding with 5% goat serum. Sections were incubated sequentially with primary and secondary antibodies, and color development was achieved with DAB. Images were captured using a digital pathology slide scanner (NanoZoomer). For IF assay, tissue slices were fixed with 4% paraformaldehyde and permeabilized using 0.1% Triton X‐100. Primary antibodies were diluted and incubated overnight, after which fluorophore‐conjugated secondary antibodies (Invitrogen, Cat# A32727) were applied. Images were captured with the LSM880 confocal microscope (Zeiss).

### SnRNA‐Seq

Brain tissues from the ipsilateral ischemic hemisphere were harvested across Sham group (*n* = 3), MCAO group (*n* = 3), and MCAO + ICA group (*n* = 3). 10× genomics single nucleus RNA sequencing was performed by Novogene Bioinformatics Technology (Beijing, China). Nuclei were isolated following the 10× genomics protocol. The efficiency of lysis and nucleus viability were assessed using trypan blue staining and a Countess II FL Automated Cell Counter. SnRNA‐seq libraries were generated using the Chromium Single Cell 3′ Library Kit v3 (Cat# 1000078; 10× genomics). For library construction, a 40 µL diluted nucleus suspension (400 nuclei µL^−1^) was combined with reverse transcription reagents and loaded into a chip for single‐cell encapsulation. The encapsulated nuclei were immediately subjected to thermal cycling to initiate reverse transcription of RNA and generate barcoded cDNA. This cDNA was then used for library construction following the standard instructions. The final libraries were measured using Qubit (Thermo Fisher Scientific), and fragment lengths were determined using a Fragment Analyzer (Advanced Analytical Technologies). Libraries were sequenced on an Illumina NovaSeq 6000 system in paired‐end mode. For each sample, FASTQ v0.20.0 was used to check the quality of the generated FASTQ data. The Cell Ranger software suite (v7.0.0) “count” pipeline was used to process the raw data and generate a gene expression matrix using default parameters. The sequencing depths (60 000 reads per cell), median unique molecular identifier count, and the percentages of mitochondrial genes were consistent across all samples.

### Integrated Analysis of Gene Expression

Data analysis was carried out using the Seurat (v4.4.0) in R environment (v4.3.1). Cell doublets were removed with DoubletFinder (v2.0.3) by optimizing the pK parameter via paramSweep_v3, with pN set to 0.25. Cells were filtered to exclude those expressing fewer than 200 or more than 6000 genes, as well as those with mitochondrial gene content exceeding 10%. Data normalization was achieved using the “LogNormalize” method, and 2000 highly variable genes were selected through the variance‐stabilizing transformation. Dimensionality reduction was carried out through principal component analysis, where significant principal components were determined based on elbow plot inflection points and JackStraw tests. Uniform manifold approximation and projection (UMAP) was utilized for clustering and visualization, and batch effects were corrected with the Harmony algorithm. Cell communities were detected with Louvain clustering, and annotations were validated against known marker genes. Differential expression analysis (Wilcoxon rank‐sum test) identified significant genes (adjusted *p*‐value < 0.05 and |log_2_FoldChange| > 0.25). Pathway enrichment analysis was conducted using clusterProfiler (adjusted *p*‐value < 0.05). Gene signatures associated with ischemic stroke were evaluated by calculating mean signature scores for each cellular subset using the AddModuleScore function in Seurat. The distribution of neuron subsets was assessed using the ratio of observed to expected cell numbers (Ro/e), with Ro/e > 1 indicating cluster enrichment under specific conditions.

### GEO Database Analysis

The peripheral blood sample datasets GSE122709 (comprising five healthy controls and ten stroke patients) and the spatial transcriptomics dataset CRA006645 (derived from the ischemic hemisphere of poststroke mouse brains) were analyzed to investigate the correlation between G3BP1 expression and cerebral ischemia.

### Human Sample Collection and Processing

A total of 30 patients with acute ischemic stroke and 30 healthy volunteers were enrolled from Anzhen Hospital in Beijing. The study was approved by the Ethics Committee of Beijing Anzhen Hospital of the Capital University of Medical Sciences (ZD2024002). Informed consent was obtained from all study participants. The diagnosis of acute ischemic stroke in patients was confirmed by MRI. Blood samples were collected from all patients prior to the administration of any therapeutic medications. Peripheral blood samples were drawn into EDTA‐anticoagulant tubes, lysed in TRIzol (Invitrogen, Cat# 15596018CN) reagents. Total RNA was isolated via using chloroform and isopropanol. The reverse transcription kit (Yeasen, Cat# 11141ES60) was used to synthesize the cDNA from RNA. qRT‐PCR amplification for the cDNA of each sample was carried out using the reagent mixtures detailed in Table S1 (Supporting Information).

### Quantification and Statistical Analysis

Statistical data were expressed as means ± standard deviation (SD). GraphPad Prism 9.0 software was used to assess statistics. Statistical differences between two groups were analyzed use a Student's *t*‐test and analysis of variance. Values of *p* < 0.05 were considered statistically significant.

## Conflict of Interest

The authors declare no conflict of interest.

## Author Contributions

L.L. and Y.‐D.G. contributed equally to this work. Conceptualization, L.L., P.‐F.T., and K.‐W.Z.; methodology, L.L., Y.‐D.G., Z.‐Y.D., Y.‐Q.W., P.‐P.Z., Z.‐Y.L., C.‐H.Z., and K.‐W.Z.; investigation, X.‐W.Z., Z.Y., Q.‐W.L., F.‐F.Z., T.‐T.W., Z.‐P.L., B.H., W.Y., and W.Z.; visualization, L.L., Y.‐D.G.; funding acquisition, K.‐W.Z.; project administration, K.‐W.Z.; supervision, P.‐F.T., C.‐H.Z., and K.‐W.Z.; writing – original draft, L.L. and K.‐W.Z.; writing – review and editing, L.L., Y.‐D.G., and K.‐W.Z.

## Supporting information



Supporting Information

## Data Availability

The data that support the findings of this study are available from the corresponding author upon reasonable request.
