# Supplementary material for: Targeting G3BP1 Condensate Topology Promotes Stress Granule Assembly via m6A‐IGF2BP1 for Ischemic Stroke Rescue
Source: Adv Sci (Weinh). 2025 Nov 20;13(6):e14703. doi: 10.1002/advs.202514703 (PMC12866838; doi:10.1002/advs.202514703)
Supplement: Supplementary file 1 — Supporting Information [file ADVS-13-e14703-s001.docx]

Supporting Information‌ for

**Targeting G3BP1 condensate topology promotes stress granule assembly via m^6^A-IGF2BP1 for ischemic stroke rescue**

*Ling Li^1, #^, Yong-Dong Guo^2, #^, Xiao-Wen* *Zhang^1^, Zhi-Yong Du^3^, Yu-Qi Wang^1^, Zhuo* *Yang^1^, Qian-Wei Luo^1^,* *Fang-Fang Zhuo^1^, Tian-Tian Wei^1^, Zheng-Ping Liu^4^, Bo Han^5^, Wei Yu^5^, Pei-Pei Zhang^6^, Wei* *Zhou^7^, Zhi-Yuan Lu^8^, Peng-Fei* *Tu^1,^ *, Chun-Hong Zheng^2,^ *, Ke-Wu Zeng^1,^ **

^1^State Key Laboratory of Natural and Biomimetic Drugs, School of Pharmaceutical Sciences, Peking University, Beijing, 100191, China.

^2^Department of Immunology, School of Basic Medical Sciences, Peking University, Beijing, 100191, China.

^3^Key Laboratory of Remodeling-Related Cardiovascular Diseases, Ministry of Education, National Clinical Research Center for Cardiovascular Diseases, Beijing Institute of Heart Lung and Blood Vessel Disease, Beijing Anzhen Hospital, Capital Medical University, Beijing, 100029, China.

^4^Shandong Engineering Research Center of New Sustained and Controlled Release Formulations and Drug Targeted Delivery Systems, Shandong Academy of Pharmaceutical Sciences, Jinan, 250101, China.

^5^School of Pharmacy/Key Laboratory of Xinjiang Phytomedicine Resource and Utilization, Shihezi University, Shihezi, 832003, China.

^6^Department of Biochemistry and Molecular Biology, School of Basic Medical Sciences, Peking University Health Science Center, Beijing, 100191, China.

^7^State Key Laboratory of Natural Medicines, School of Traditional Chinese Pharmacy, China Pharmaceutical University, Nanjing, 211198, China.

^8^ School of Pharmaceutical Sciences & Institute of Materia Medica, Shandong First Medical University & Shandong Academy of Medical Sciences, Jinan, 250117, China.

^#^These authors contributed equally.

*Corresponding author. e-mail: [pengfeitu@bjmu.edu.cn](mailto:pengfeitu@bjmu.edu.cn) (PF Tu), chunhong_zheng @bjmu.edu.cn (CH Zheng), and ZKW@bjmu.edu.cn (KW Zeng)


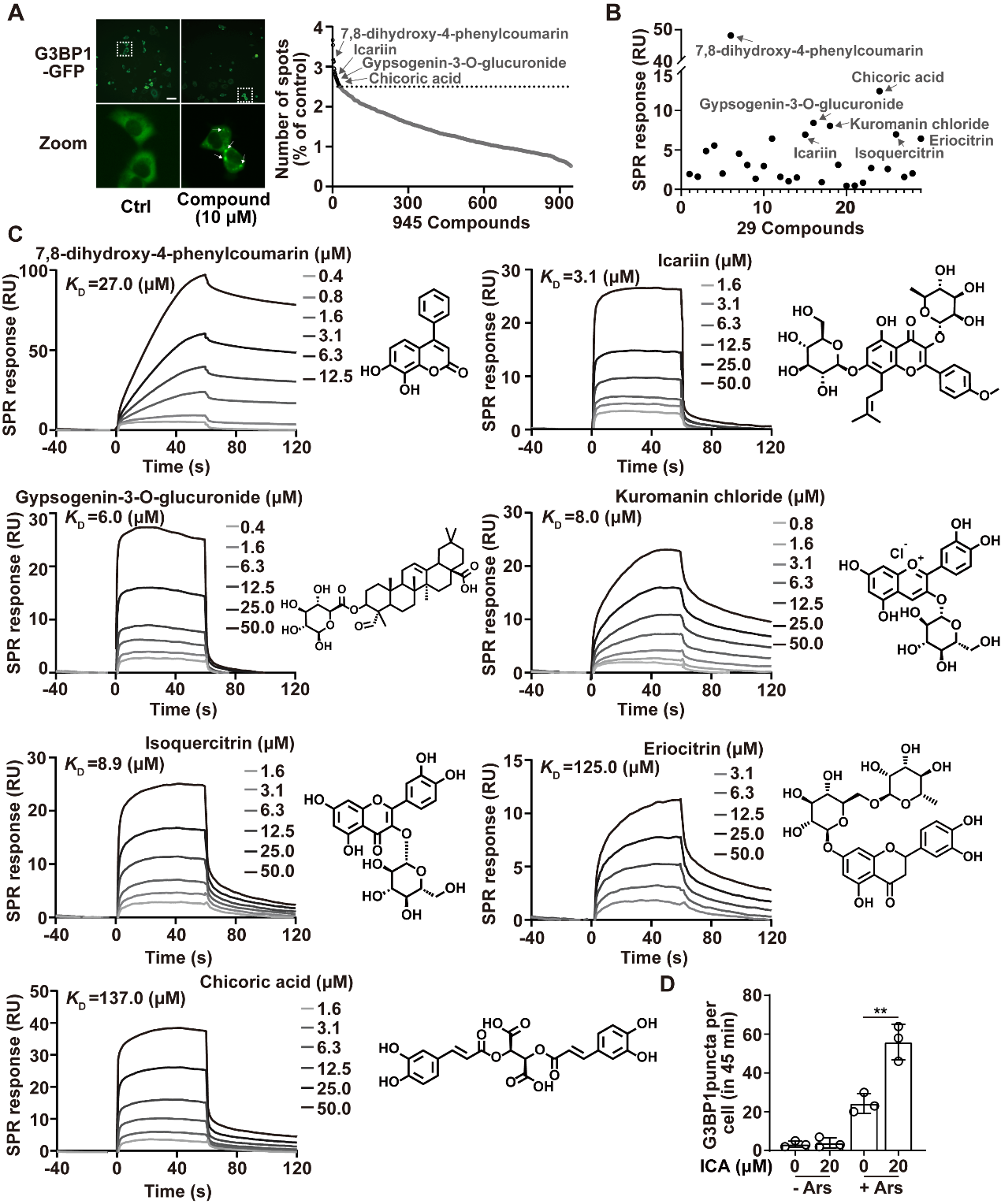


Figure S1. Systematic screening identifies ICA as a G3BP1 phase separation inducer. A) High content screening of 945 compounds from chemical library in HEK293T cells transfected with G3BP1-GFP plasmids. The percent of control for fluorescent spots was plotted with the compounds (bar: 20 μm). B) The SPR response of 29 compounds was evaluated through SPR screening at a concentration of 50 μM. C) The SPR analysis was performed to investigate the binding affinity of the top 7 compounds with G3BP1 in a concentration-dependent manner. D) The statistical chart that U2OS cells overexpressed G3BP1-GFP were exposed to low-dosage arsenite (100 μM) with or without ICA (20 μM) treatment. * *p* < 0.05, ** *p* < 0.01.


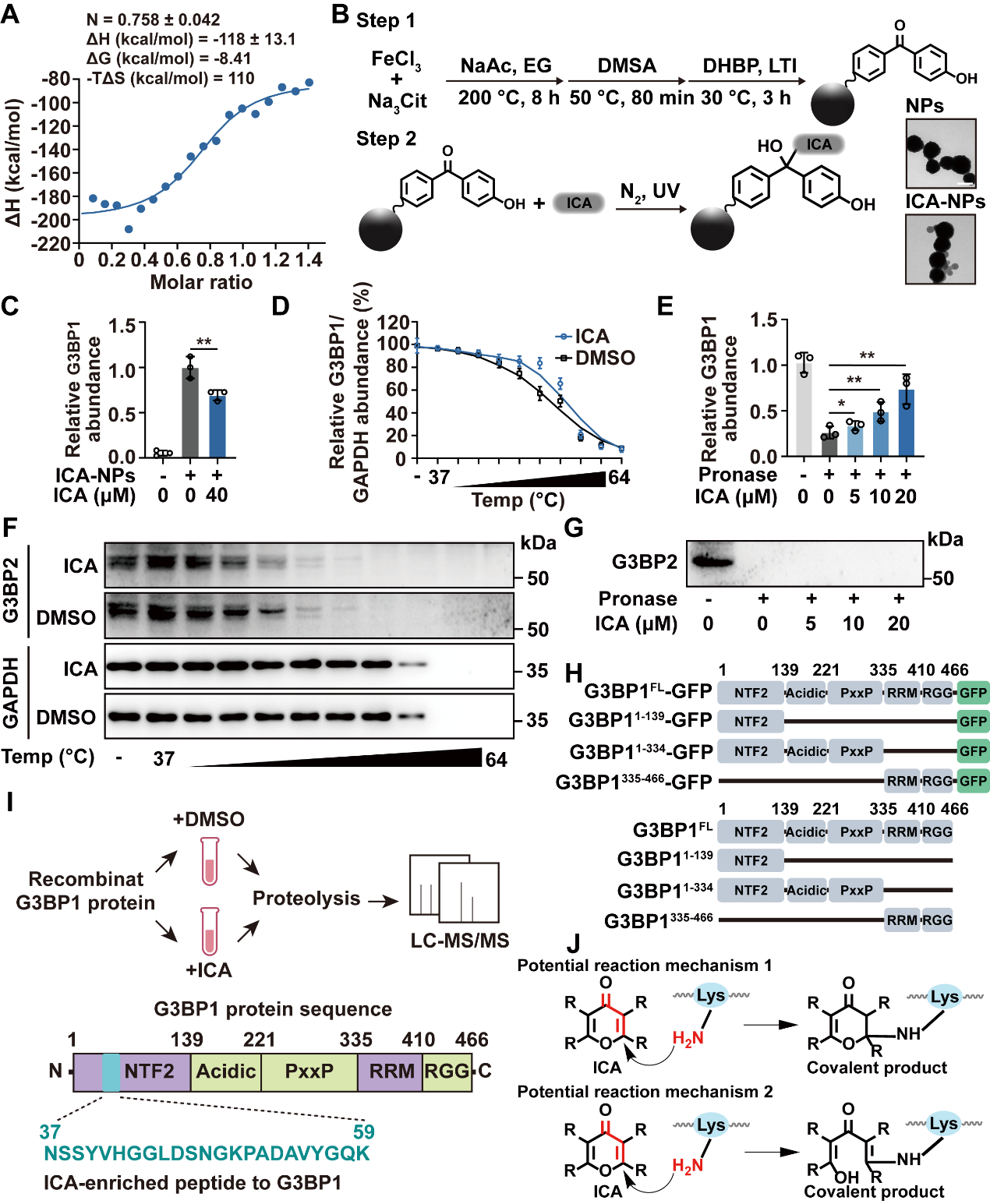


Figure S2. ICA directly binds to the NTF2L domain of G3BP1. A) ITC analysis of ICA binding to recombinant G3BP1 protein. B) The two-step preparation of nanoparticles (NPs) and ICA- cross-linked nanoparticles (ICA-NPs). And the TEM image of NPs and ICA-NPs (bar: 100 nm). C) The statistical chart of pull-down analysis that ICA binding to G3BP1 in cell lysate. D) The statistical chart that ICA promoted the temperature-dependent stabilization of G3BP1 through CETSA. E) The statistical chart that ICA enhanced G3BP1 resistance against pronase through DARTS. F) CETSA analysis to detect the interaction between ICA and G3BP2. G) DARTS analysis to analyze the interaction between ICA and G3BP2. H) Diagram of the establishment of different G3BP1 truncations with or without the GFP tag. I) The workflow for identifying ICA-binding peptides using LC-MS/MS analysis, as well as the peptides enriched with ICA. J) The potential reaction mechanism for the covalent binding of ICA to lysine residues. Data are expressed as the mean ± SD for 3 individual experiments. * *p* < 0.05, ** *p* < 0.01.


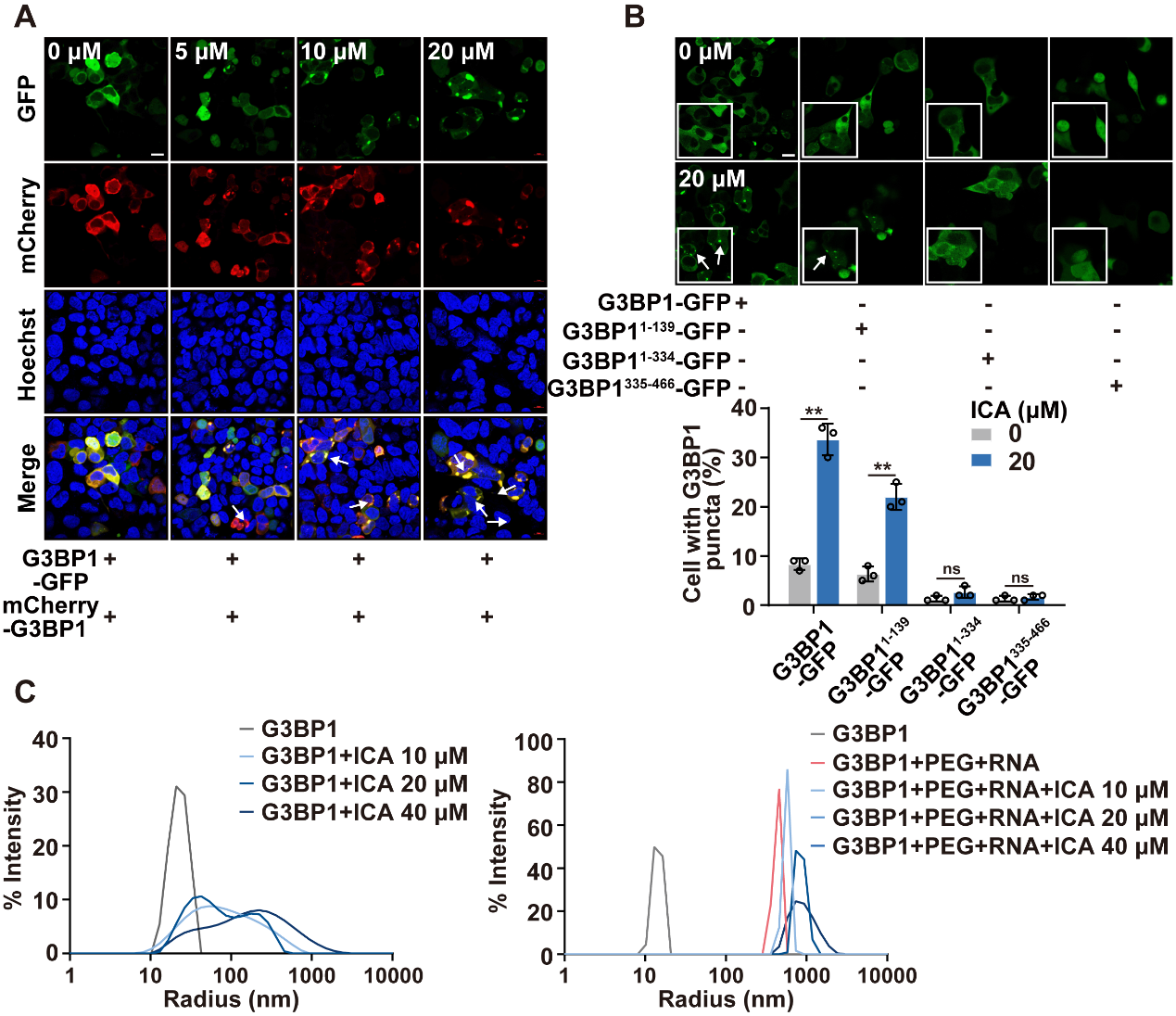
 Figure S3. ICA induces G3BP1 phase separation via the NTF2L domain dimerization. A) The HEK293T cells were co-transfected with G3BP1-GFP and mCherry-G3BP1 and then exposed to 5, 10, and 20 μM ICA (bar: 20 μm). B) The HEK293T cells transfected with different GFP-G3BP1 variants plasmids and treated with ICA (bar: 20 μm). C) Hydrodynamic radius distributions of G3BP1 protein measured by dynamic light scattering (DLS) in the absence or presence of ICA, with or without RNA and PEG. Data are expressed as the mean ± SD for 3 individual experiments. * *p* < 0.05, ** *p* < 0.01; ns no significance.


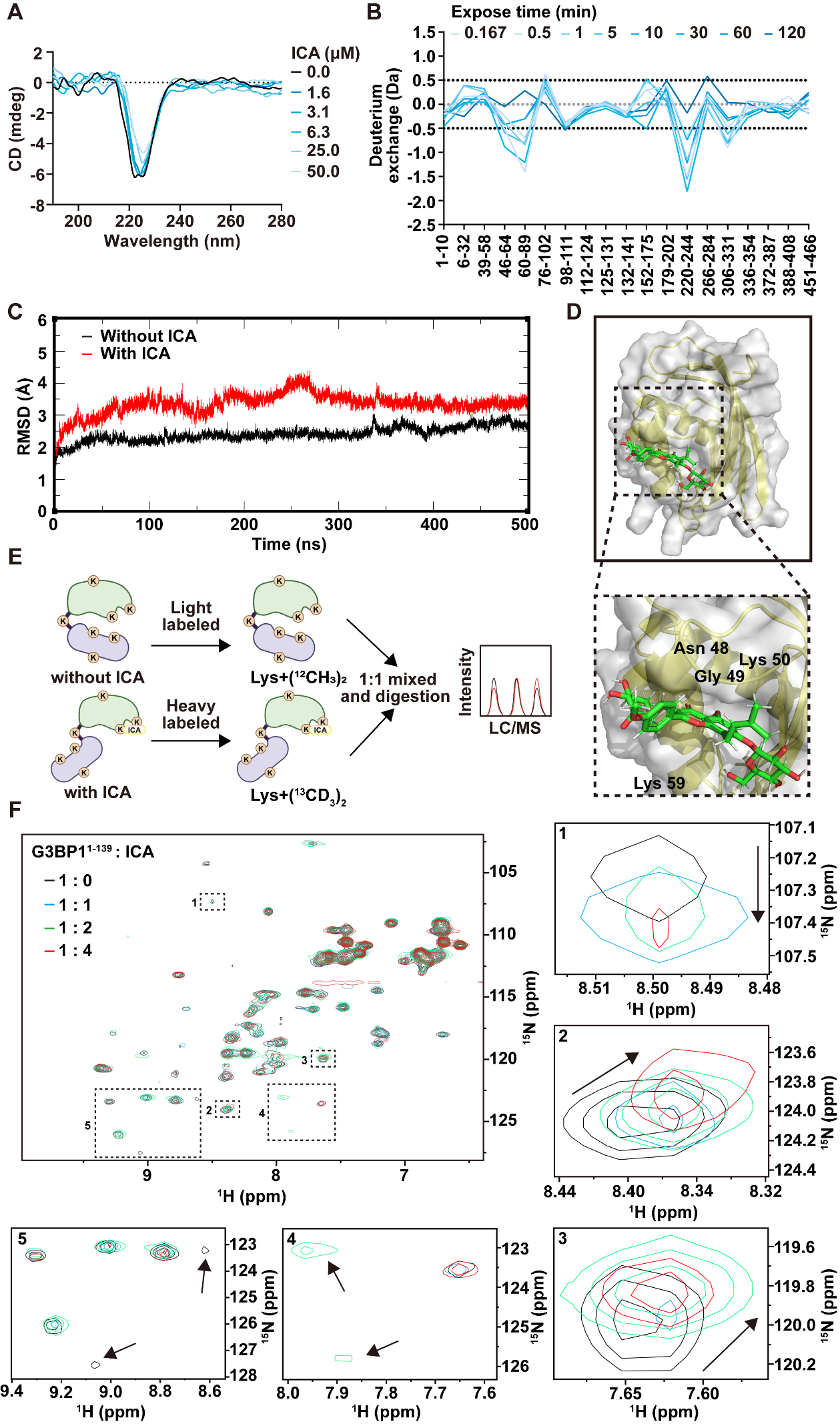


Figure S4. Allosteric regulation drives the LLPS of G3BP1 through a ‘close-to-open’ pattern. A) CD spectra analysis for ICA-induced G3BP1^1-139^ conformational change. B) HDX analysis the different deuterium exchange of per peptide in G3BP1 after 0.167, 0.5, 1, 5, 10, 30, 60, and 120 min to exchangeable amides. C) Root mean square deviation (RMSD) for molecular dynamics (MD) of G3BP1^1-139^ with ICA (PDB: 8v1l). D) Molecular docking identified the binding pocket of ICA within the G3BP1^1-139^ protein. E) The process of quantitative lysine reactivity profiling strategy. F) ^1^H-^15^N HSQC spectra of G3BP1^1-139^ and treatment with various concentrations of ICA. The molar ratio of G3BP1^1-139^ to ICA respectively 1:0 (black), 1:1 (blue), 1:2 (green), and 1:4 (red).


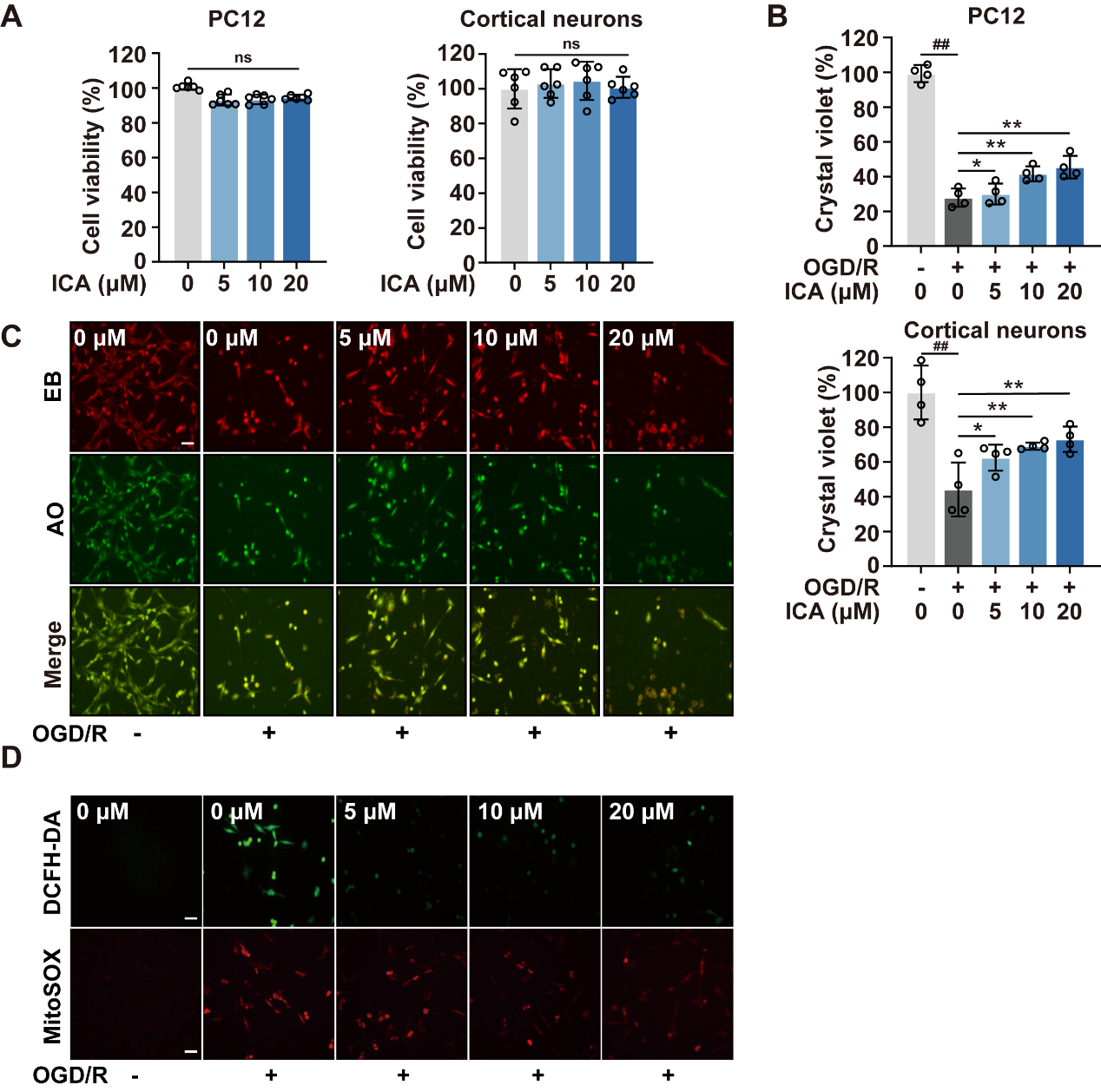


Figure S5. ICA demonstrates a cell-protective capability against various stress injuries. A) Cell viability of PC12 cells and cortical neurons exposed to ICA (5, 10, and 20 μM) using the MTT assay. B) The statistical results of crystal violet staining following OGD/R induction and ICA (5, 10, and 20 μM) treatment. C) ICA decreased OGD/R-induced PC12 cells apoptosis by AO/EB staining. (bar: 50 μm). D) The DCFH-DA and MitoSOX red staining for detecting the intracellular hydrogen peroxide and mitochondrial superoxide anion (bar: 50 μm). Data are expressed as the mean ± SD for 3-6 individual experiments. * *p* < 0.05, ** *p* < 0.01; ^#^ *p* < 0.05, ^##^*p* < 0.01. ns no significance.


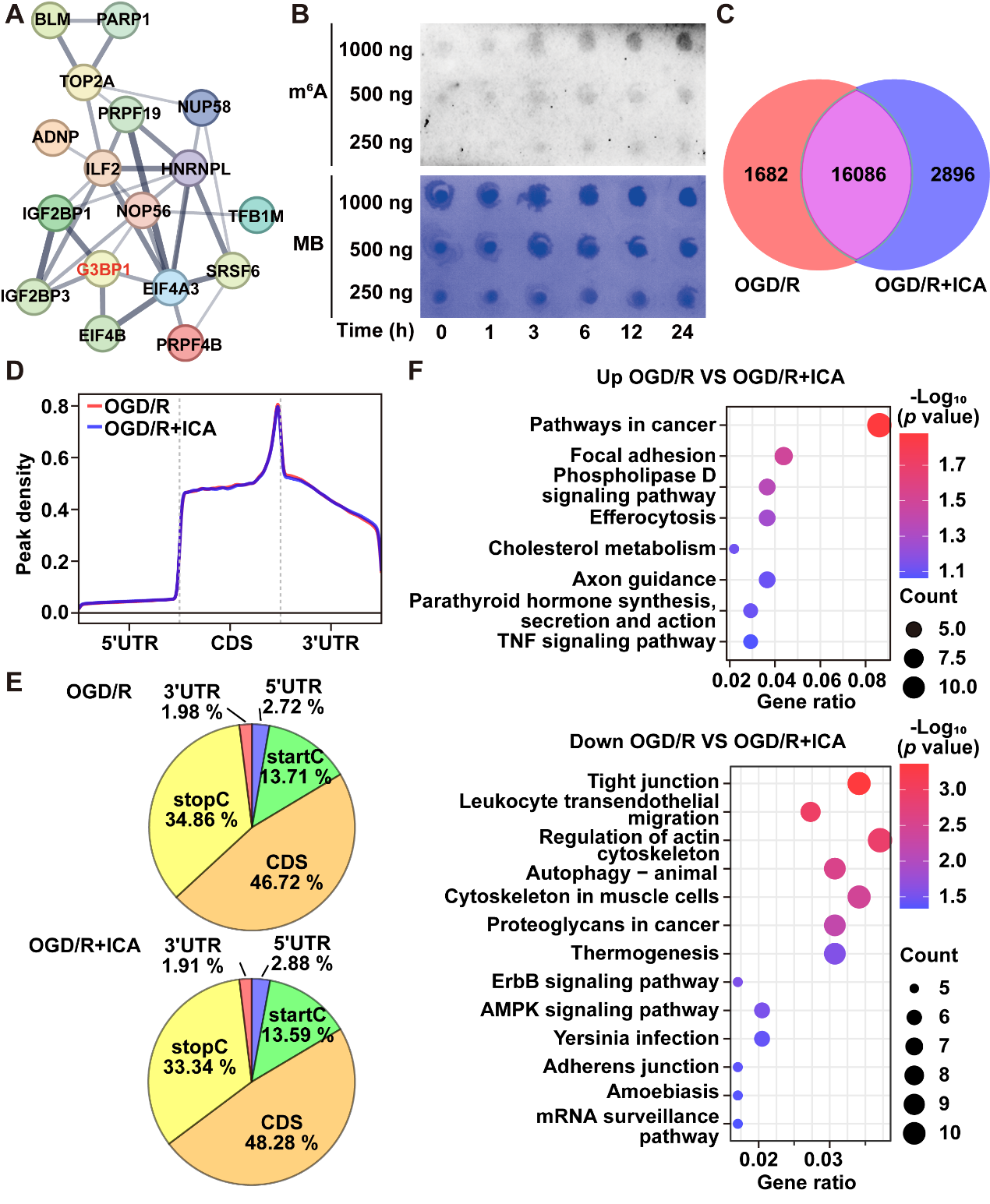


Figure S6. G3BP1 condensates recruit IGF2BP1 to modulate RNA stability in m^6^A-dependent manner. A) Protein-protein interactions (PPI) analysis of the increased interactions with G3BP1 following ICA treatment. B) The dot blot assay for the time-dependent effect of ICA treatment on the global m^6^A abundance. C) Venn diagram of m^6^A-modified genes in OGD/R group (n=3) and OGD/R+ICA (n=3) group. D) Density of differential m^6^A peaks along transcripts, containing five parts: 5′UTR, CDS, 3′UTR, startC, and stopC. E) Pie charts presented the region of m^6^A peaks in OGD/R group and OGD/R+ICA group. F) The KEGG analysis for the up or down m^6^A peaks in OGD/R group compared with OGD/R+ICA group.


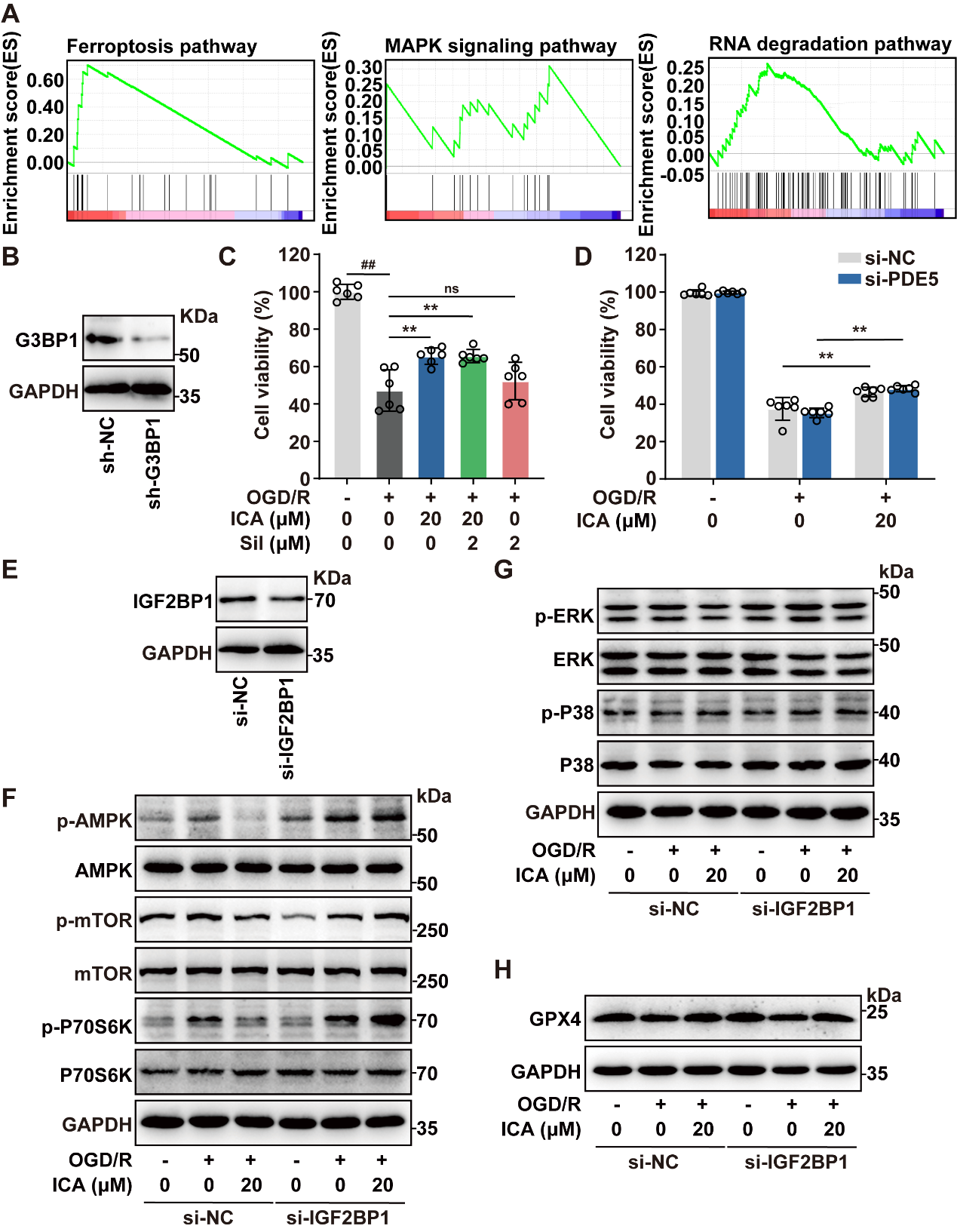
 Figure S7. G3BP1 regulates neuronal survival via AMPK-MAPK-GPX4 signaling axis. A) GSEA of differentially expressed genes upon ICA treatments and OGD/R as control. B) Western blot analyzed for the levels of G3BP1 protein after sh-G3BP1 lentivirus transfection. C) Cell viability of PC12 cells exposed to ICA or Sil using the MTT assay following OGD/R induction. D) MTT assay detected the cell viability after PDE5 knockdown with or without ICA treatment follow by OGD/R. E) Western blot analyzed the levels of IGF2BP1 protein after IGF2BP1 knockdown. F-H) Western blot analysis of p-AMPK, AMPK, p-mTOR, mTOR, p-P70S6K, P70S6K, p-P38, P38, p-ERK, ERK, and GPX4 protein expression after IGF2BP1 knockdown with or without ICA treatment during OGD/R. GAPDH served as a loading control. Data are expressed as the mean ± SD for 3-6 individual experiments. **p* < 0.05, ** *p* < 0.01; ^#^ *p* < 0.05, ^##^ *p* < 0.01; ns no significance.


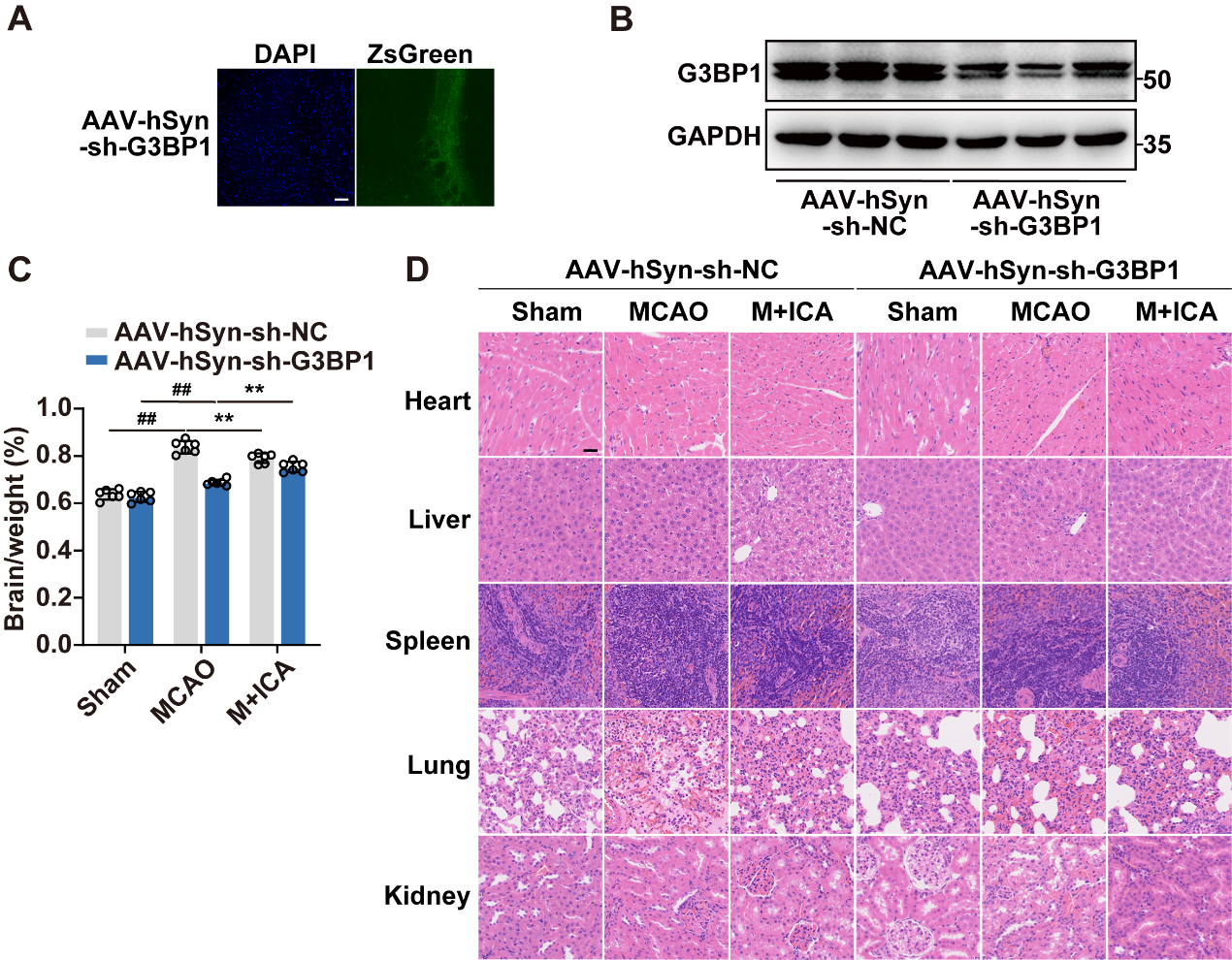


Figure S8. G3BP1 as a therapeutic target in the middle cerebral artery occlusion (MCAO) rat model. A) Fluorescence imaging to assess the fluorescence intensity in tissue after AAV-hSyn-sh-G3BP1 stereotaxic injection into the brain (bar: 50 μm). B) Western blot analyzed the levels of G3BP1 protein after AAV-hSyn-sh-G3BP1/NC stereotaxic injection into the brain. C) The brain/weight of different groups. Sham (n = 6), MCAO (n = 6), and MCAO+ICA (n = 6). D) H&E staining detected the morphology of the heart, liver, spleen, lung, and kidney (bar: 20 μm). Data are expressed as the mean ± SD for 3-6 individual experiments. * *p* < 0.05, ** *p* < 0.01; ^#^ *p* < 0.05, ^##^*p* < 0.01.


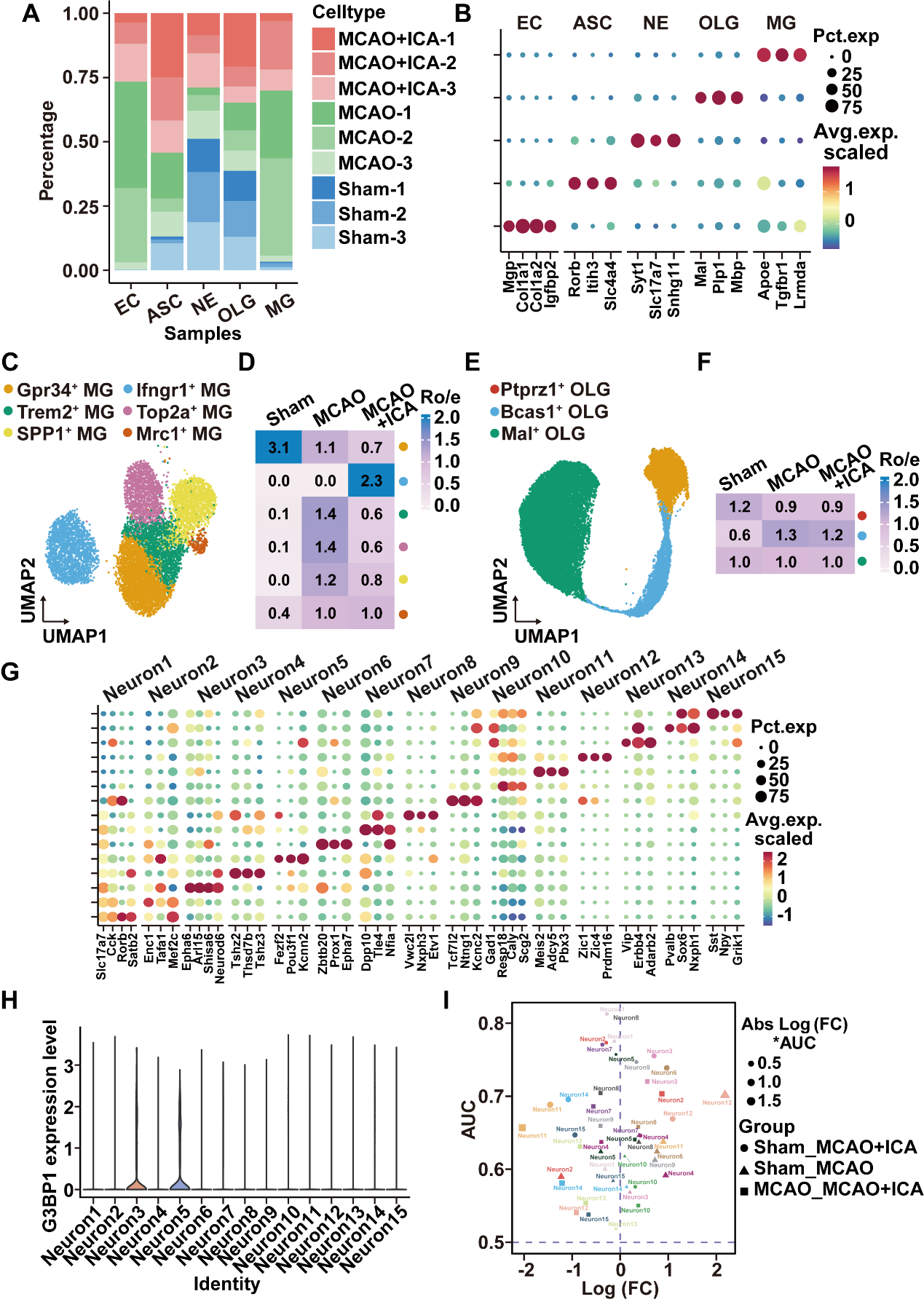


Figure S9. G3BP1 represents a biomarker with translational medical value for ischemic stroke. A) The tacked bar plot depicting the relative abundance of each cell cluster within each group. Sham (n = 3), MCAO (n = 3), and MCAO+ICA (n = 3). B) The dotplots depicting curated marker genes for all major cell clusters. The intensity of the dot color represents the average expression of this module in a given cluster relative to the other clusters. The size of the dot reflects the percentage of cells that express the specified module. C) UMAP represented the subcluster analysis of the Microglia. D) Heatmap showing the prevalence of Microglia subcluster. E) UMAP represented the subcluster analysis of the Oligodendrocyte. F) Heatmap showing the prevalence of Oligodendrocyte subcluster. G) The dotplots depicting curated marker genes in neuron subtypes. The intensity of the dot color represents the average expression of this module in a given subtype relative to the other subtypes. The size of the dot reflects the percentage of cells that express the specified module. H) Violin plots demonstrated the expression; level of G3BP1 within neuron subpopulations. I) The relative AUC of fold change for each subtype.


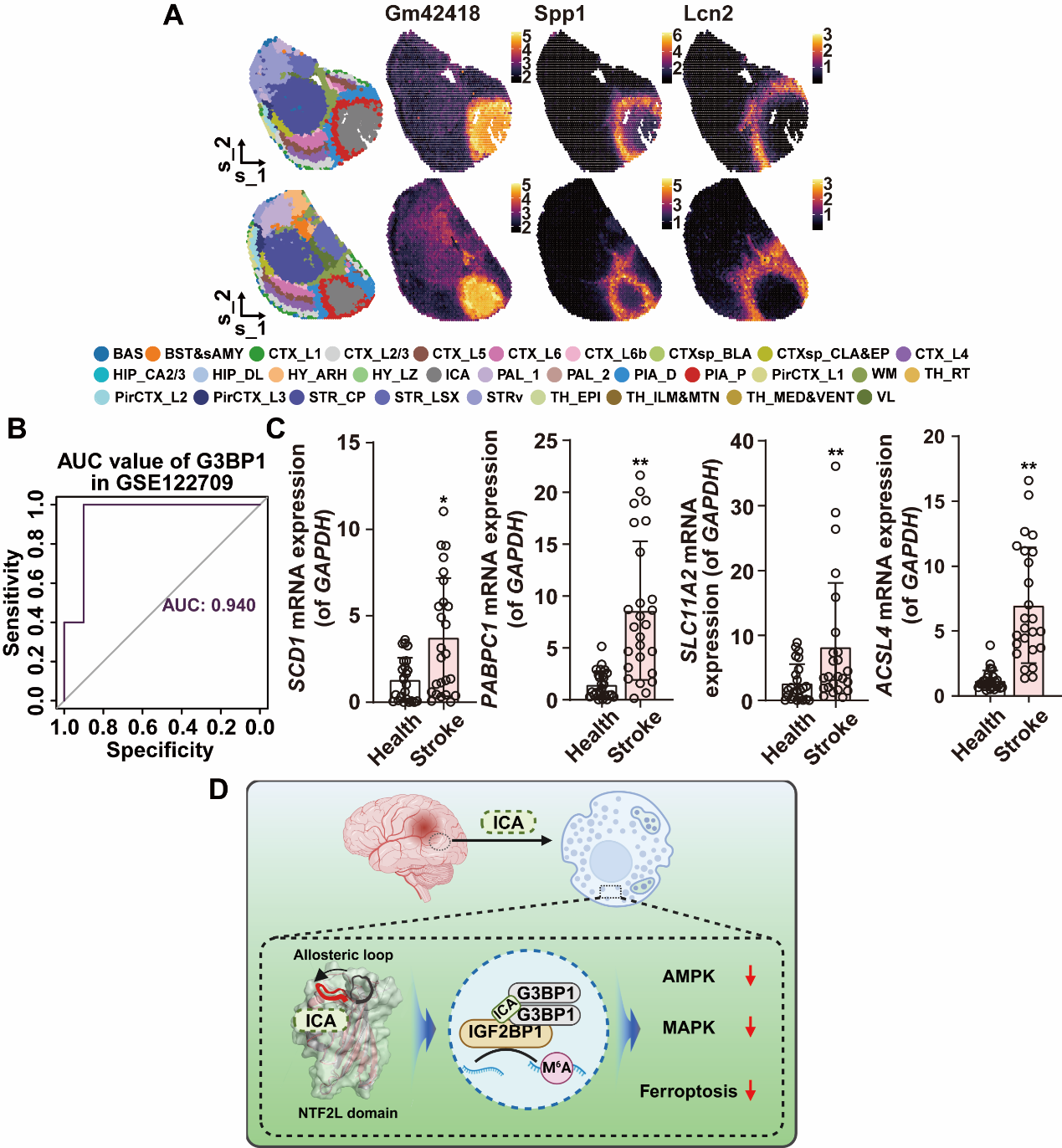


Figure S10. G3BP1 represents a biomarker with translational medical value for ischemic stroke. A) Spatial transcriptomics visualization revealed the regional expression patterns of the genes Gm42418 in the infarct core area (ICA), Spp1 in the proximal region of the peri-infarct area (PIA_P), and Lcn2 in the distal region of the peri-infarct area (PIA_D) across distinct brain ischemia regions. The color intensity corresponds to the magnitude of gene expression. B) AUC analyzes the expression level of G3BP1 in the peripheral blood sample of healthy controls and stroke patients. C) The mRNA expression of *SCD1*, *PABPC1*, *SLC11A2*, and *ACSL4* in PBMCs from patients with and without acute cerebral ischemic stroke (30 patients with acute ischemic stroke and 30 healthy volunteers). D) ICA demonstrated G3BP1-dependent therapeutic efficacy against cerebral ischemia-reperfusion injury. Data are expressed as the mean ± SD for at least 3 individual experiments. * *p* < 0.05, ** *p* < 0.01.

**Table 1. Primer sequences for quantitative RT-PCR analysis.**

| **Gene** | **Sequence** |
| --- | --- |
| *Scd1-F* | 5’-TTCCTACCTGCAAGTTCTACACC-3’ |
| *Scd1-R* | 5’-CCGAGCTTTGTAAGAGCGGT-3’ |
| *Pabpc1-F* | 5’-TCCACTTTTGCGAAGTGATG-3’ |
| *Pabpc1-R* | 5’-CTACGCGTATGTGAACT-3’ |
| *Acsl4-F* | 5’-TCTTCTCCGCTTACACTCTCT-3’ |
| *Acsl4-R* | 5’-CTTATAAATTCTATCCATGATTTCCGGA-3’ |
| *Slc11a2-F* | 5’-TGCTGGGTCCTGAACAGAAG-3’ |
| *Slc11a2-R* | 5’-GCAACTTAAATCCAGCCACTG-3’ |
| *G3bp1-F* | 5’-CTTTGGTGGGTTTGTCACTG-3’ |
| *G3bp1-R* | 5’-TGCTGTCTTTCTTCAGGTTCC-3’ |
| *GAPDH-F* | 5’-ATGAATGGGCAGCCGTTAGG-3’ |
| *GAPDH-R* | 5’-CAGAGTTAAAAGCAGCCCTGG-3’ |
| *R-GAPDH-F* | 5’-TGGTGAAGGTCGGTGTGAAC-3’ |
| *R-GAPDH-R* | 5’-AACTTGCCGTGGGTAGAGTC-3’ |
| *R-Mknk2-F* | 5’-TCGTTCAAGGGGCAGAATCC-3’ |
| *R-Mknk2-R* | 5’-CTTCTTGGCGTCTGGGATGT-3’ |
| *R-Mtor-F* | 5’-ACCCATCCAACCTGATGCTG-3’ |
| *R-Mtor-R* | 5’-TCGAGACCGGTAACCTCCA-3’ |
| *R-Map2k7-F* | 5’-AGGATCGACCTCAACCTGGA-3’ |
| *R-Map2k7-R* | 5’-AGGAGCAGGGCTTAGAGTGA-3’ |
| *R- Pde5 -F* | 5’-TGCCCTTTGGAGACAAAACG-3’ |
| *R- Pde5 -R* | 5’-GGTCGGTCAAATTCAGAGGC-3’ |
